# Magnesium Structure‐Function Integration Platform for Spatiotemporal Multi‐Modality Therapy: Combining Hormonotherapy and Immunotherapy in Prostate Cancer

**DOI:** 10.1002/advs.202515235

**Published:** 2025-12-23

**Authors:** Rui Zan, Qianping Mao, Keyi Wang, Guodong Zou, Shi Yang, Hua Qiu, Xiang Fang, Guiqing Wang, Xinyi Zhou, Jiexia Wen, Shuai Jiang, Ran Huang, Qiuming Peng, Tao Suo

**Affiliations:** ^1^ Department of Biliary Surgery Zhongshan Hospital & College of Biomedical Engineering & Yiwu Research Institute Fudan University Shanghai 200032 P. R. China; ^2^ Yiwu Research Institute of Fudan University Yiwu 322000 P. R. China; ^3^ State Key Laboratory of Metastable Materials Science and Technology Yanshan University Qinhuangdao 066004 P. R. China; ^4^ College & Hospital of Stomatology Key Laboratory of Oral Diseases Research of Anhui Province Anhui Medical University Hefei 230032 P. R. China; ^5^ School of Mechanical and Automotive Engineering Shanghai University of Engineering Science Shanghai 201620 P. R. China

**Keywords:** androgen deprivation therapy, immunogenic cell death, multi‐modality therapy, spatiotemporal response, structure‐function integration

## Abstract

Metal‐based immunotherapy represents a promising strategy for enhancing antitumor efficacy; however, its clinical application is limited by challenges such as inefficient drug delivery, low specificity, and suboptimal therapeutic outcomes. In this study, a therapeutic platform is constructed on a magnesium (Mg) surface utilizing self‐healing thiolated hyaluronic acid (HA‐SH) and cell membrane‐derived vesicle (CMV) drug system, which improves structural stability, enables spatiotemporal drug release, and facilitates immune‐hormone combination therapy for prostate cancer. CMV with phospholipid bilayers promotes HA‐SH disulfide bond interactions through weak hydrophobic interactions, mitigating corrosion and ensuring structural integrity. Additionally, HA‐SH exhibits glutathione (GSH)‐responsive drug release within the tumor microenvironment. CMV facilitates pH‐sensitive drug delivery and enables efficient cytoplasmic entry via membrane fusion mechanisms, ensuring precise spatiotemporal control. Through drug library screening, ginsenoside Rb1 is identified as a key therapeutic agent, competitively inhibiting androgen receptor signaling. Coupled with hydrogen release from Mg, it induces immunogenic cell death and promotes the formation of tertiary lymphoid structures (TLS) via inhibiting the PI3K‐AKT pathway, achieving synergistic androgen deprivation therapy and immune activation. This implantable drug delivery system effectively tackles mechanical stability, local drug delivery, and systemic immune activation concerns, demonstrating substantial translational promise for various malignancies to improve treatment effectiveness and reduce structural failure risks.

## Graphical abstract

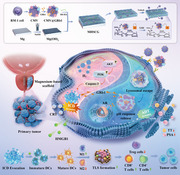

## 1 Introduction

In the field of oncology, metallic immunotherapy has recently emerged as a promising therapeutic strategy,^[^ ^1^, ^2^ ^]^ particularly with the attention directed toward bioactive metallic implants known for their structural support and ability to release local metal ions.^[^ ^3^, ^4^ ^]^ Among these, magnesium (Mg) alloys, recognized for their bioactive properties, have been extensively studied in clinical settings for various applications such as bone screws, luminal stents, and oral repair membranes.^[^ ^5^, ^6^ ^]^ These alloys have shown promise in exerting anti‐tumor effects by releasing Mg ions and hydrogen gas (H_2_), which possess notable immunomodulatory properties.^[^ ^7^, ^8^ ^]^ However, achieving optimal anti‐tumor efficacy with Mg alloy implants may require compromising certain mechanical support characteristics, potentially jeopardizing structural integrity. Consequently, the development of Mg alloy implants that can simultaneously provide structural support and anti‐tumor functionality poses a complex challenge in the realm of tumor therapy.

To tackle these challenges, employing functional coatings has arisen as a promising approach, encompassing techniques such as micro‐arc oxidation, layered double hydroxide, and polymer embedding chemotherapeutic agents.^[^ ^9^, ^10^ ^]^ These coatings prolong the implantation lifespan and enhance the efficacy of cancer therapy.^[^ ^11^ ^]^ Nevertheless, this passive system exhibits constraints in maintaining long‐term structural integrity and regulating drug release kinetics. Hyaluronic acid (HA), a key component of ureteral mucosa, is commonly used in biomimetic coating technology. HA modified with sulfhydryl (‐SH) demonstrates self‐repair and drug loading properties via a reversible dynamic disulfide bond exchange reaction.^[^ ^12^ ^]^ Importantly, disulfide bonds can be cleaved by high glutathione (GSH) concentrations in the tumor microenvironment, enabling stimulus‐responsive drug release.^[^ ^13^, ^14^ ^]^ This self‐repairing controlled‐release coating maintains structural integrity and enables precise therapeutic function regulation, offering a novel approach for coating design.

Androgen deprivation therapy (ADT) is a well‐established postoperative strategy for managing prostate cancer (PCa).^[^ ^15^ ^]^ However, the immunogenicity of traditional androgen antagonists often leads to complications and treatment resistance, posing a significant clinical challenge.^[^ ^16^ ^]^ Natural products offer promise in PCa treatment due to their multi‐target mechanisms and biocompatibility.^[^ ^17^ ^]^ Ginsenosides, active compounds isolated from ginseng, possess hormonal therapeutic properties by competitively inhibiting androgen receptors (AR), thereby inducing androgen deprivation.^[^ ^18^, ^19^ ^]^ Cell membrane‐derived vesicles (CMV) serve as an effective vehicle for encapsulating ginsenosides, enhancing drug targeting and bioavailability, which is attributed to improved receptor‐mediated endocytosis through surface receptors and chemotactic homing capabilities, avoiding lysosomal transport and enabling direct drug release into the cytoplasm.^[^ ^20^, ^21^ ^]^ Recently, emerging evidence underscores the promising clinical efficacy of combining ADT with immunotherapy in the treatment of PCa, warranting further exploration in this therapeutic avenue.^[^ ^22^ ^]^

**Figure 1:**
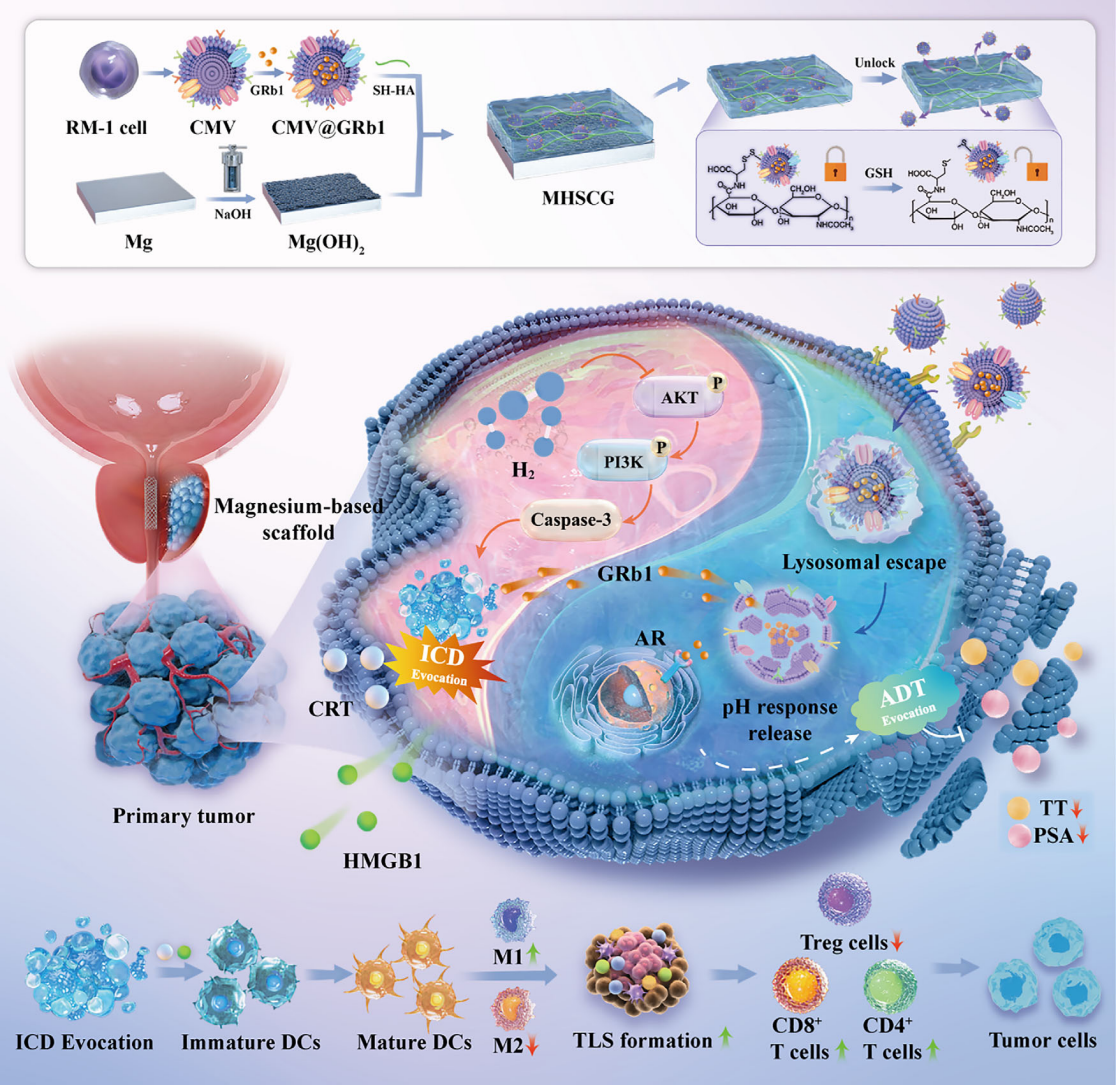
The schematic illustration of the MHSCG platform for spatiotemporal responsive GRb1 release to induce androgen deprivation therapy (ADT) and immunogenic cell death (ICD) multimodal therapy in prostate cancer.

In this study, we developed a therapeutic system (MHSCG) comprising CMV‐encapsulated ginsenoside loaded onto a Mg surface with HA‐SH for dual protection of the substrate and combined ADT with immunization against PCa (**Figure** **1**). The HA‐SH side‐chain‐linked phospholipid bilayer facilitates the oriented polymerization of HA via disulfide bond exchange reactions, leading to a dynamic anti‐corrosion effect. Within the tumor microenvironment (TME), GSH cleaves the disulfide bond, triggering the responsive release of the drug, which is then transported to the cytoplasm through the chemotaxis effect of CMV. The drug ginsenoside Rb1 (GRb1), identified through a drug library screening, is released from CMV in response to the acidic pH of the tumor, facilitating a sequential reaction for precise spatiotemporal treatment. Furthermore, H_2_ generated from the Mg substrate prompts the release of damage‐associated molecular patterns (DAMPs) from the tumor, leading to dendritic cell maturation and T cell activation, thereby inducing immunogenic cell death (ICD) in PCa. This investigation lays the groundwork for advanced biodegradable implants that combine structural integrity with comprehensive oncological therapy. The synergistic actions of Mg‐based immunomodulation, drug‐targeted delivery, and multi‐pronged oncological therapy address critical challenges in PCa treatment, offering an innovative approach to managing both local and systemic tumor progression.

## 2 Results and Discussion

### 2.1 Preparation, Characterization, and Responsiveness of MHSCG

**Figure 2:**
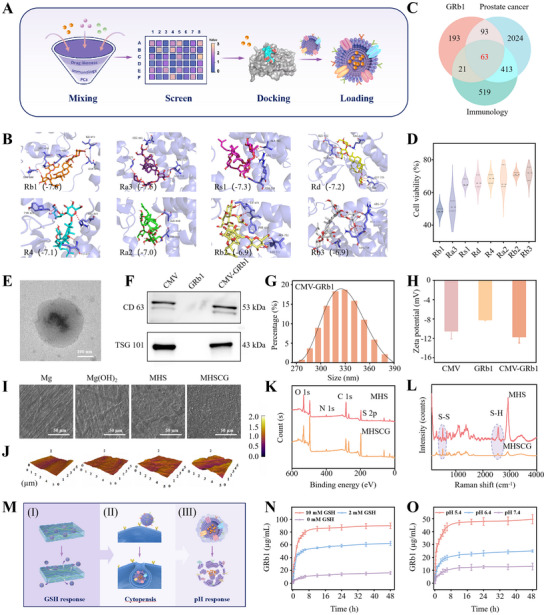
Virtual drug screening, preparation, and characterization of the MHSCG platform. A) Schematic illustration of the virtual drug screening process and the preparation of cell membrane‐derived vesicles (CMV) encapsulated ginsenoside. B) Prediction of the binding energy of the androgen receptor (AR) with eight kinds of ginsenosides (Rb1, Ra3, Rs1, Rd, R4, Ra2, Rb2, Rb3). C) Venn diagram depicting the intersection of genes associated with GRb1, prostate cancer, and immunology. D) Cell viability of prostate cancer RM‐1 cells following 24 h treatment with the eight ginsenosides. E) Transmission electron microscopy (TEM) image of CMV‐GRb1. F) Protein expressions of CD63 and TSG101 in CMV were determined by Western blot. G) Size distribution of the CMV‐GRb1, H) Zeta potential of the CMV, GRb1, and CMV‐GRb1, respectively. I) The surface morphology of Mg, Mg(OH)_2_, MHS, and MHSCG samples. J) Atomic force microscopy (AFM) images of the different samples. K,L) X‐ray photoelectron spectroscopy (XPS) (K) and Raman spectroscopy analysis (L) of the MHS and MHSCG platform. M) Schematic diagram of responsive release of MHSCG. N,O) Release profiles of GRb1 under different concentrations of GSH (N) and pH (O) conditions. ^*^ *p*< 0.05, ^**^ *p*< 0.01, and ^***^ *p*< 0.001.

To obtain drugs with PCa therapeutic effect, we collected a library of ginsenosides with antitumor and ADT for screening. Drug likeness (DL) and oral bioavailability (OB) are often used to screen compounds that are unlikely to be drugs. The threshold was set to DL >0.18 with OB ≤ 8% as the limit of the active ingredient. According to the screen, 45 kinds of ginsenoside were screened out (**Figure** **2**A). Subsequently, the interaction between AR and ginsenoside was studied by the molecular docking technique. As shown in Figure 2B, the binding sites of ginsenoside and AR form appropriate spatial complementarity, and the binding energy of ginsenoside and AR was quantitatively analyzed, which selected the top eight representative ginsenosides. The molecular docking score of GRb1 and AR was the lowest, indicating that these ginsenosides and AR have a strong binding affinity. Based on the strong interaction between GRb1 and AR, we hypothesized that GRb1 binds to AR through hydrogen bonds, etc., and antagonizes androgen binding to AR to achieve the ADT therapeutic effect. By searching GeneCards and DisGeNET databases, 2593 PCa‐related disease genes, 1016 immunology‐related genes, and 370 GRb1 regulatory genes were obtained, respectively (Figures 2C; S1, Supporting Information). The predicted targets of GRb1 crossed with those of PCa and immunology‐related diseases, and 63 common targets were identified as potential targets of GRb1 in PCa with immunology therapy. Furthermore, we demonstrated that GRb1 has a better tumor inhibitory effect than other ginsenoside compounds by cell proliferation experiments, highlighting the potential therapeutic role of GRb1 in PCa (Figure 2D). CMVs are composed of lipid bilayers with diverse bioactive proteins on their surfaces and are commonly utilized for therapeutic agent delivery.^[^ ^23^ ^]^ In this study, PCa CMV was isolated using gradient centrifugation. Subsequently, GRb1 was loaded into the CMV using an extruder to produce spherical drug‐loaded therapeutic particles, as depicted in Figure 2E. The presence of membrane markers such as CD63 and TSG101 facilitated the interaction between drug‐loaded vesicles and tumor cells (Figure 2F). The average diameter of the loaded vesicular nanoparticles was 331.5 ± 30.5 nm (Figure 2G), with a zeta potential of −12.13 ± 1.04 mV (Figure 2H). In comparison, drug‐free cells exhibited vesicle diameters of 284.1 ± 32.1 nm and zeta potentials of −10.14 ± 0.91 mV.

Subsequently, we developed the MHSCG therapeutic system on a Mg surface using HA‐SH and drug‐loaded vesicles. Scanning electron microscopy (SEM) results showed successful coating of MHSCG onto Mg surface at a thickness of ≈5 µm, with uniform distribution of drug‐loaded vesicles (Figures 2I; S2, Supporting Information). Atomic force microscopy (AFM) indicated higher surface roughness of MHSCG compared to MHS and Mg coatings (Figure 2J). X‐ray photoelectron spectroscopy (XPS) results demonstrated a decrease in S2p, O1s, and Cls peaks of MHSCG relative to MHS, suggesting the involvement of sulfhydryl (S‐H) in HA‐SH during drug coupling (Figures 2K; S3, Supporting Information). This finding was further supported by Raman spectroscopy, which showed a significant reduction in the S‐H peak of MHSCG, indicating efficient loading of drug‐loaded vesicles by HA‐SH (Figure 2L).

Next, the drug‐responsive release characteristics of MSHCG were examined (Figure 2M). Previous research has demonstrated that GSH in the TME can cleave the disulfide bonds present in HA‐SH, leading to drug release.^[^ ^24^ ^]^ This study focused on investigating the drug release profile of MSHCG under varying GSH concentrations. The findings indicated that MSHCG exhibited notable stability. At a GSH concentration of 0 mM, the drug release rate was markedly slow, attributed to the disulfide‐anchored drug structure that prevented premature drug release commonly observed with conventional coatings.^[^ ^25^ ^]^ With increasing GSH concentrations, a responsive drug release from MSHCG was observed (Figure 2N). Subsequently, the GRb1 were delivered from drug‐loaded vesicles in a cascade manner in response to the mildly acidic tumor environment (Figure 2O).

### 2.2 MHSCG Enhances the Structural Stability of Mg in the Urethral Environment

**Figure 3:**
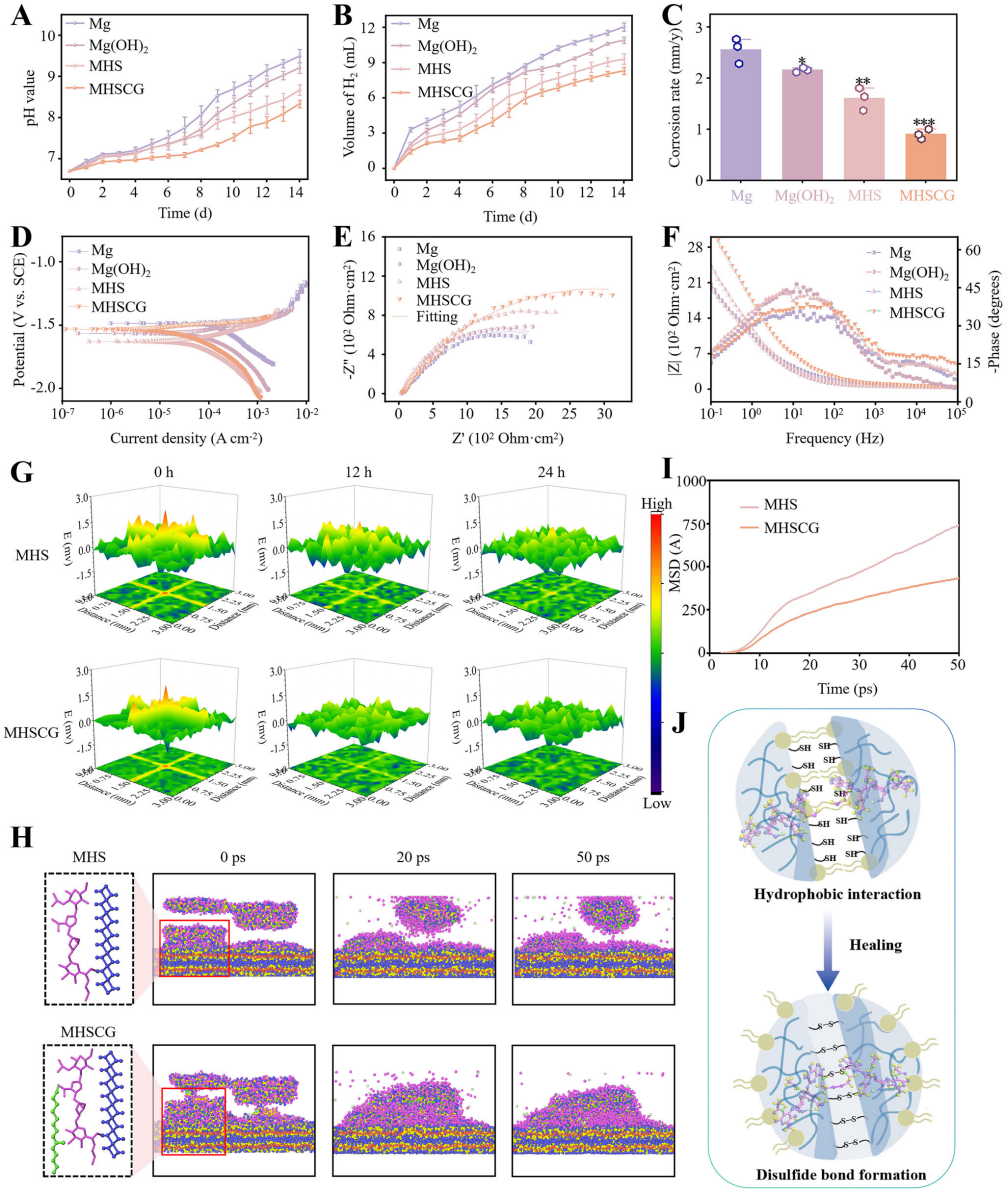
Structural stability of Mg protected by MHSCG. A) The pH variation and B) the hydrogen evolution volume of different samples immersed in artificial urine over a 14‐day period. C) Corrosion rates of the samples during the 14‐day immersion. D) Tafel polarization curves, E) Nyquist plots, and F) Bode plots of the samples immersed in artificial urine for 14 h, analyzed by electrochemical tests. G) Scanning vibrating electrode technique (SVET) monitoring of corrosion current density on the surfaces of MHS and MHSCG after immersion at 0, 12, and 24 h. H) Molecular dynamics (MD) simulation of the self‐healing performance of the MHS and MHSCG platforms. I) Quantitative analysis of the mean square displacement (MSD) curves derived from MD simulations. J) Schematic illustration of the self‐healing mechanism of the MHSCG platform. *p< 0.05, **p< 0.01, and ***p< 0.001.

Rapid degradation leading to structural failure is a primary issue causing failure in Mg devices.^[^ ^26^ ^]^ Surface coating technology plays a crucial role in prolonging the lifespan of Mg implants.^[^ ^27^ ^]^ However, conventional coatings face challenges related to insufficient stability and complex preparation procedures. Here, an MHSCG system was developed for the Mg surface prevention. Subsequent immersion of the samples in artificial urine demonstrated that the MHSCG samples displayed exceptional stability over a 72 h period without any observable fractures (Movie S1, Supporting Information). **Figure** **3**A showed the monitoring pH values in the immersion solution revealed a gradual increase, with the pH value reaching 8.33 ± 0.09 after 14 days, which was significantly lower than that of the naked Mg group (9.49 ± 0.16). Furthermore, minimal H_2_ release was detected from the MHSCG sample during the 14‐day immersion, indicating superior performance compared to other degradation products (Figure 3B). As illustrated in Figure 3C, the corrosion rate of the Mg substrate was measured at 0.90 ± 0.10 mm y^−1^, which was lower than that of naked Mg (2.55 ± 0.24 mm y^−1^), Mg(OH)_2_ (2.16 ± 0.04 mm y^−1^), and MHS (1.60 ± 0.22 mm y^−1^), which further confirmed by diffusion of chloride ions through the MHSCG and Mg(OH)_2_ coatings (Figure S4, Supporting Information).

Electrochemical experiments were conducted to assess the initial protective efficacy of MHSCG on Mg substrates. The instantaneous corrosion rate (*CR*) was determined by the formula as follows:

(1) $$\mathrm{CR}=3270\times \frac{M\cdot I_{\mathrm{corr}}}{\mathrm{Vd}}$$

where *M* represents the atomic mass, *V* denotes the valence, and *d* signifies the density of the substrate.^[^ ^28^ ^]^ Lower corrosion currents (*I_corr_)* indicate superior protection.

The hierarchy of *I_corr_* values observed was Mg >Mg(OH)_2_ >MHS >MHSCG, with MHSCG exhibiting the lowest instantaneous corrosion rate of 0.55 mm y^−1^ among all groups (Figure 3D; Table S1, Supporting Information). Nyquist plots revealed that MHSCG displayed a broader arc diameter in a medium‐frequency environment, indicative of enhanced charge transfer between MHSCG and artificial urine, thereby impeding the ingress of corrosion ions into the substrate (Figure 3E). This observation was further corroborated by the Bode plot, with MHSCG demonstrating the highest impedance modulus at low frequencies (Figures 3F; S5 and Table S2, Supporting Information). The low‐frequency impedance modulus of MHSCG exhibited a progressive increase over time, signifying an improvement in corrosion resistance.

Based on the aforementioned results, we speculated that MHSCG exhibits self‐healing properties attributed to dynamic disulfide bonds from HA‐SH, with CMV enhancing this self‐healing capability. Subsequently, we assessed the self‐healing efficacy of MHS and MHSCG utilizing the scanning vibrating electrode technique (SVET). Our findings revealed that, following a 24 h immersion in artificial urine, MHSCG demonstrated superior self‐healing kinetics and extent compared to MHS (Figure 3G), a phenomenon corroborated through DFT simulation. As shown in Figure 3H, at 50 ps, the impaired MHSCG coating exhibited directed polymerization induction facilitated by CMV, resulting in a lower mean square displacement (MSD) relative to freely diffusing MHS (Figure 3I). This process led to a more uniform surface coverage, manifesting a pronounced self‐healing effect that effectively safeguards the integrity of the substrate. The phospholipid bilayer structure of CMV on the HA‐SH surface promotes disulfide bond interaction through weak hydrophobic interaction, thus promoting healing (Figure 3J).

### 2.3 The MHSCG Inhibits the Malignant Biological Behavior of PCa Cells In Vitro

**Figure 4:**
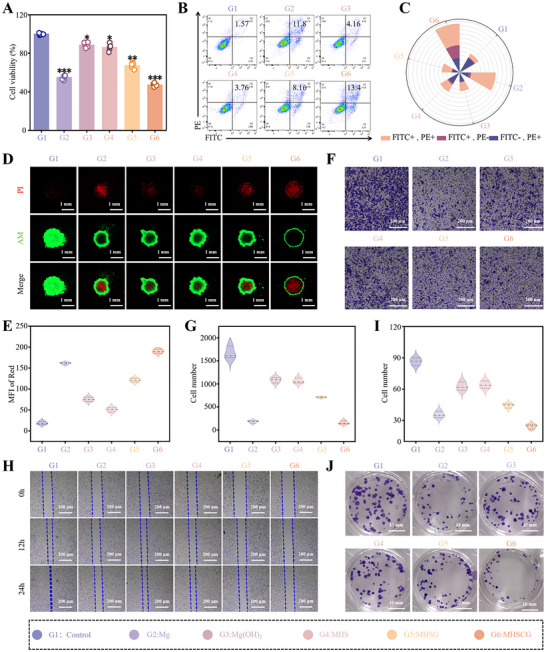
In vitro evaluation of the anti‐tumor efficacy of MHSCG. A) Cell viability of RM‐1 cells treated with different samples for 24 h. B) Apoptosis analysis of RM‐1 cells by flow cytometry and C) quantitative assessment of the apoptosis rate. D) Live/dead staining of RM‐1 cell spheroids and E) quantification of dead cells (stained red). F) Cell invasion assay and G) quantitative analysis of the invasive capacity of RM‐1 cells. H) Migration area of RM‐1 cells assessed by scratch assay at 0, 12, and 24 h, respectively. I,J) Colony formation assay and corresponding quantification of cell colonies. ^*^ *p*< 0.05, ^**^ *p*< 0.01, and ^***^ *p*< 0.001 (n ≥ 3).

**Figure** **4** evaluated the cytological impact of MHSCG on PCa RM‐1 cells in vitro. The cell viability assay using the cell counting kit‐8 (CCK‐8) revealed a substantial decrease in RM‐1 cell viability when treated with MHSCG, resulting in a 52.72 ± 1.56% inhibition rate, slightly higher than the 45.01 ± 2.05% inhibition observed in the Mg group (Figure 4A). The apoptosis rate exhibited a similar inhibitory pattern, with MHSCG showing the highest efficacy, followed by Mg, MHSG, Mg(OH)_2_, MHS, and Control (Figure 4B,C). Moreover, CMV‐encapsulated GRb1 demonstrated enhanced bio‐efficacy compared to unmodified GRb1. Live‐dead cell staining of cell spheroids confirmed that CMV facilitated the intracellular delivery of GRb1, leading to tumor cell death (Figure 4D,E). Subsequently, the inhibition of MHSCG on cell migration, invasion, and proliferation was assessed. MHSCG significantly reduced invasive cell counts from 1671 ± 109 to 160 ± 29, hindered cell migration, covering only 30% of the distance after 24 h compared to complete migration in the Control groups (Figures 4F–H; S6, Supporting Information). Colony formation assays showed that cells treated with MHSCG formed colonies at a mere 28.08% of the levels observed in the Control group, effectively suppressing RM‐1 cell proliferation (Figure 4I,J). MHSCG restrained cell migration to neighboring tissues and markedly inhibited tumor colony growth. Remarkably, MHSCG exhibited robust structure‐function integration, effectively suppressing tumor malignancy to a level comparable to that of the uncoated Mg group while enhancing the stability of the Mg substrate.

### 2.4 MHSCG Induces ADT and ICD Multimodal Therapy In Vitro

**Figure 5:**
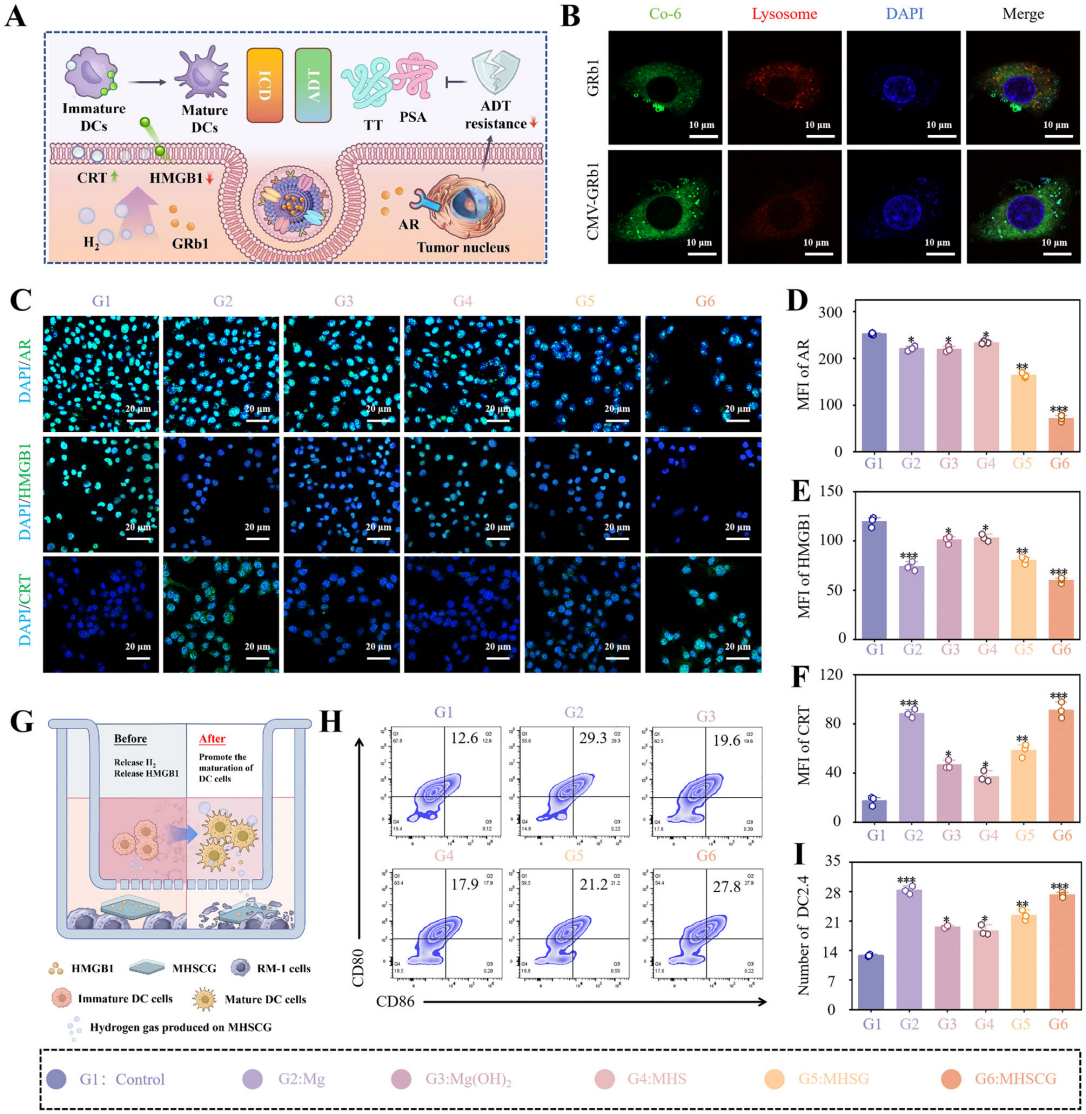
MHSCG‐induced multimodal therapy combining ADT and ICD in vitro. A) Schematic illustration of the in vitro anti‐tumor mechanism of MHSCG. B) Cellular uptake (stained green) and lysosomal escape (stained red) of GRb1 and CMV‐GRb1 in RM‐1 cells observed by confocal laser scanning microscopy (CLSM). C) CLSM images showing the expression of ADT‐related androgen receptor (AR), ICD‐related high‐mobility group box 1 (HMGB1), and calreticulin (CRT) under different treatments. D–F) Quantitative analysis of AR (D), HMGB1 (E), and CRT (F) expression in RM‐1 cells under various treatments. G) Schematic diagram of activated dendritic cell (DCs) with the MHSCG treatment. H) Flow cytometry analysis of DCs maturation. I) Quantitative assessment of DCs maturation in RM‐1 cells under different treatments. ^*^ *p*< 0.05, ^**^ *p*< 0.01, and ^***^ *p*< 0.001 (n ≥ 3).

The CMV drug‐delivery system was chosen to enhance cell surface fusion and prevent drug degradation in endo‐lysosomes, thereby increasing drug bioavailability (**Figure** **5**A).^[^ ^29^, ^30^ ^]^ Confocal laser scanning microscopy (CLSM) analysis of GRb1 (stained with coumarin 6, green) and endo‐lysosomes (stained with LysoTracker Red, red) revealed that CMV‐encapsulated GRb1 penetrated more cells compared to untreated GRb1, with minimal lysosomal signaling observed (Figures 5B; S7, Supporting Information). As shown in Figure 5C,D, this CMV‐mediated membrane fusion delivery of GRb1 significantly reduced androgen receptor (AR) expression, counteracts androgens, and accomplishes androgen deprivation therapy (ADT). Furthermore, degradation products from the MHSGH system triggered immunogenic cell death (ICD) by releasing DAMPs containing high mobility group box 1 (HMGB1) and calreticulin (CRT).^[^ ^31^, ^32^ ^]^ CRT facilitates phagocytosis by immature dendritic cells (DCs) and macrophages, promoting the maturation of immature DCs into mature DCs. HMGB1 translocated from the nucleus to the extracellular space, serving as a biomarker associated with ICD in tumor cells. These components act as immune adjuvants crucial for recruiting and maturing antigen‐presenting cells (APCs), thereby enhancing immune activation.^[^ ^33^ ^]^ Immunofluorescence and real‐time PCR (RT‐qPCR) findings demonstrated that MHSGH could upregulate CRT expression and induce HMGB1 release, with the immunomodulatory effects primarily attributed to the synergistic action of H_2_ and GRb1 (Figures 5E,F; S8, Supporting Information).

The immunoactivation capacity of MHSCG was investigated using the Transwell assay, with PC1 cells seeded in the upper chamber and DC2.4 cells in the lower chamber (Figure 5G). Flow cytometry analysis revealed a significant increase in the proportion of CD80^+^ CD86^+^ cells to 27.17 ± 0.49% with MHSCG treatment, compared to 12.83 ± 0.21% in the Control group, indicating enhanced maturation of DC cells (Figure 5H,I). These findings indicated that the MHSCG system has the potential to enhance the therapeutic efficacy of drugs and elicit multimodal therapeutic effects in PCa by modulating hormone levels and stimulating immune responses through the release of GRb1 and H_2_.

### 2.5 The Anti‐Tumor Efficacy of MHSCG In Vivo

**Figure 6:**
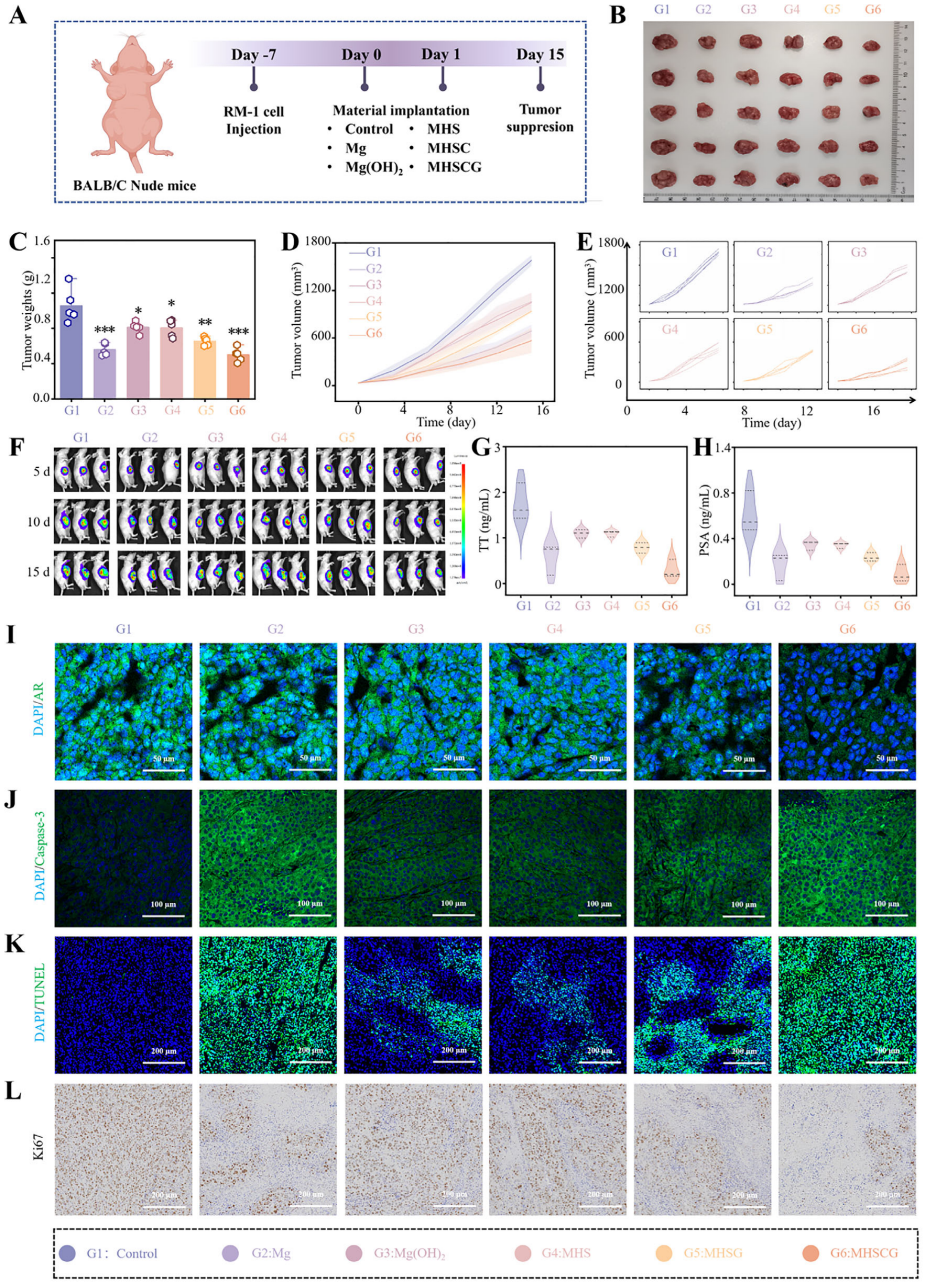
The antitumor efficacy of MHSCG in vivo. A) Schematic illustration of the treatment schedule in Balb/c nude mice. B) Digital photographs of tumors after 15 d of treatment. C) Tumor weights were measured after 15 d of treatment. D,E) Tumor volume growth curves for different treatment groups (D) and individual tumor growth curves (E) in nude mice over 15 d. F) Bioluminescence imaging of tumor‐bearing mice at 5 d, 10 d, and 15 d post‐treatment. G,H) Serum levels of total testosterone (TT) (G) and prostate‐specific antigen (PSA) are related to androgen receptor level in vivo. (H). I–L) CLSM images of tumor tissues stained for androgen receptor (AR) (I), caspase‐3 (J), TUNEL (K), and Ki67 (L). ^*^ *p*< 0.05, ^**^ *p*< 0.01, and ^***^ *p*< 0.001.

A nude mouse model of prostate cancer was established by subcutaneously injecting RM‐1 cells to evaluate the in vivo antitumor efficacy of MHSCG samples (**Figure** **6**A). Once tumors reached ≈60 mm^3^ in size, RM‐1‐luc tumor‐bearing mice were randomly allocated into six groups: 1) Control; 2) Mg; 3) Mg(OH)_2_; 4) MHS; 5) MHSG; and 6) MHSCG. Tumor volume was monitored over a 15‐day treatment period, with tumor weight assessed at 15 days. MHSCG displayed the most potent tumor inhibitory effect among the six groups, leading to a 50% reduction in tumor weight compared to the Control group (Figures 6B,C; S9, Supporting Information). The tumor growth curve further confirmed the significant in vivo inhibitory effect of MHSCG, as evidenced by a decelerated tumor volume increase. Specifically, the final tumor volume in the MHSCG group was markedly lower at 567.75 ± 157.15 mm^3^ compared to the Control group's volume of 1580.99 ± 67.20 mm^3^, indicating substantial tumor growth suppression (Figure 6D,E). Then, fluorescence imaging of tumor‐bearing mice revealed a notable decrease in fluorescence intensity of MHSCG compared to both the Control and Mg(OH)_2_ groups at 15 d post‐treatment, suggesting effective in vivo therapeutic outcomes of MHSCG (Figures 6F; S10, Supporting Information). Mice serum was subsequently collected for androgen‐related assessment. Blood biochemical analyses showed decreased levels of total testosterone (TT) and prostate‐specific antigen (PSA) expression in the MHSCG group, suggesting that it exerts a superior effect on androgen deprivation therapy (ADT) in vivo (Figure 6G,H).^[^ ^34^ ^]^ Additionally, tumor tissues were subjected to immunofluorescence staining, revealing the lowest androgen receptor (AR) expression in the MHSCG group (Figure 6I). Histological analyses, including caspase‐3, terminal deoxynucleotidyl transferase dUTP nick labeling (TUNEL), and Ki‐67 staining of tumor sections, indicated increased apoptosis/necrosis and decreased proliferation in tumor cells of the MHSCG group, leading to the activation of the apoptosis cascade in tumors (Figures 6J–L; S11, Supporting Information).

### 2.6 MHSCG Promotes Antitumor Immunity In Vivo

**Figure 7:**
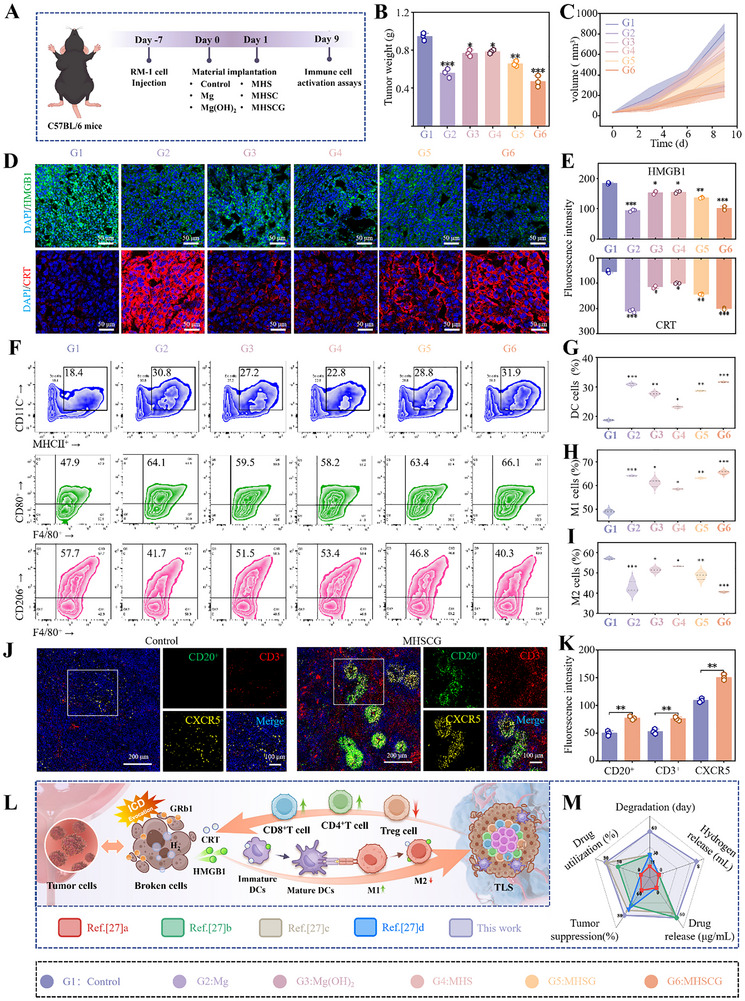
In vivo antitumor immunity induced by MHSCG. A) Schematic illustration of the treatment schedule in C57BL/6 mice. B) Tumor weights were measured after 9 d of treatment. C) Tumor volume growth curves for different treatment groups in C57BL/6 mice over 9 d. D) CLSM images of ICD‐related markers, HMGB1, and CRT in tumor tissues after 9 d of treatment. E) Quantitative analysis of the fluorescence intensity of HMGB1 and CRT expression. F) Flow cytometric analysis of DCs, M1 macrophage cells, and M2 macrophage cells in tumor tissues by various treatments. G–I) Quantitative assessment of DCs, M1, and M2 cells in tumor tissues based on flow cytometric analysis. J,K) Representative CLSM images (J) and quantitative analysis of TLS‐related markers, CD20^+^ B cells (green), CD3^+^ T cells (red), CXCR5 (yellow), and DAPI (blue) in the tumor after 9 d of treatment. L) Schematic diagram of MHSCG immune activation in vivo. M) Comparative analysis of the antitumor efficacy and degradation performance of the MHSCG platform with previous studies.^[^ ^37^, ^38^, ^39^, ^40^ ^] *^ *p*< 0.05, ^**^ *p*< 0.01, and ^***^ *p*< 0.001 (n ≥3).

**Figure** **7**A aimed to elucidate the underlying in vivo antitumor immunity of MHSCG, given its efficient antitumor effect. A tumor‐bearing model was established in C57 mice to assess the therapeutic efficacy (Figures 7B,C; S12 and S13, Supporting Information). Immunofluorescence staining of tumor tissues revealed that the MHSCG group exhibited enhanced surface CRT exposure and HMGB1 release (Figure 7D,E). As shown in Figure 7F,G, flow cytometry analysis indicated that the MHSCG group facilitated the maturation of lymphocyte DCs, consistent with in vitro DC maturation results, suggesting MHSCG's ability to induce ICD. Recent research has highlighted the crucial role of immunosuppressive cells within the tumor microenvironment, such as T regulatory cells (Tregs) and immune‐related inflammatory cells, in facilitating tumor immune evasion.^[^ ^35^ ^]^ To investigate the potential of the MHSCG system in modulating the immunosuppressive microenvironment through immune checkpoint inhibitors (ICDs), we conducted a detailed analysis of Treg cells, M1, and M2. Our findings revealed that the MHSCG group exhibited the lowest Treg cell infiltration rate (34.2 ± 0.43%) within tumors and significantly enhanced the M1/M2 ratio in tumor tissues (Figures 7H,I; S16, Supporting Information). With the M1 macrophage polarization, the secretion of inflammatory cytokines (IFN‐γ, IL‐7, and TNF‐α, Figure S14, Supporting Information) and chemokines (CXCL13, CXCR5, CXCL10 and CCL19, Figure S15, Supporting Information) in tumor tissues regulates the maturation of T lymphocytes and B cells within the tumor microenvironment, facilitating the formation of tertiary lymphoid structures (TLS). Moreover, immunofluorescence analysis revealed an accumulation of CD20^+^ B cells, CD3^+^ T cells, and chemokine CXCR5 in tumors treated with MHSCG (Figure 7J,K), initiating TLS formation to boost anti‐tumor immunity induced by ICD.^[^ ^36^ ^]^ The analysis of tumor infiltration immunity via flow cytometry demonstrated a significant increase in CD8^+^ T cells by MHSCG treatment (1.16 fold higher than the Control group), with a similar trend observed for CD4^+^ T cells (2.81 fold higher than the Control group) (Figure S16, Supporting Information). These results demonstrate that the MHSCG treatment can stimulate in vivo immune responses and remodel the immunosuppressive tumor microenvironment, thereby converting the tumor immunophenotype from “cold” to “hot” (Figure 7L).

The study compared the anti‐tumor efficacy, degradation kinetics, drug utilization efficiency, drug release profiles, and hydrogen release of the MHSCG system with those of existing stents, based on their stent degradation and anti‐tumor outcomes (Figure 7M).^[^ ^37^, ^38^, ^39^, ^40^ ^]^ The findings demonstrated that the MHSCG system exhibited enhanced structural support and overall performance in tumor therapy, offering a potential model for advancing prostate stent technology in the future.

### 2.7 Molecular Biological Mechanism of MHSCG to PCa

**Figure 8:**
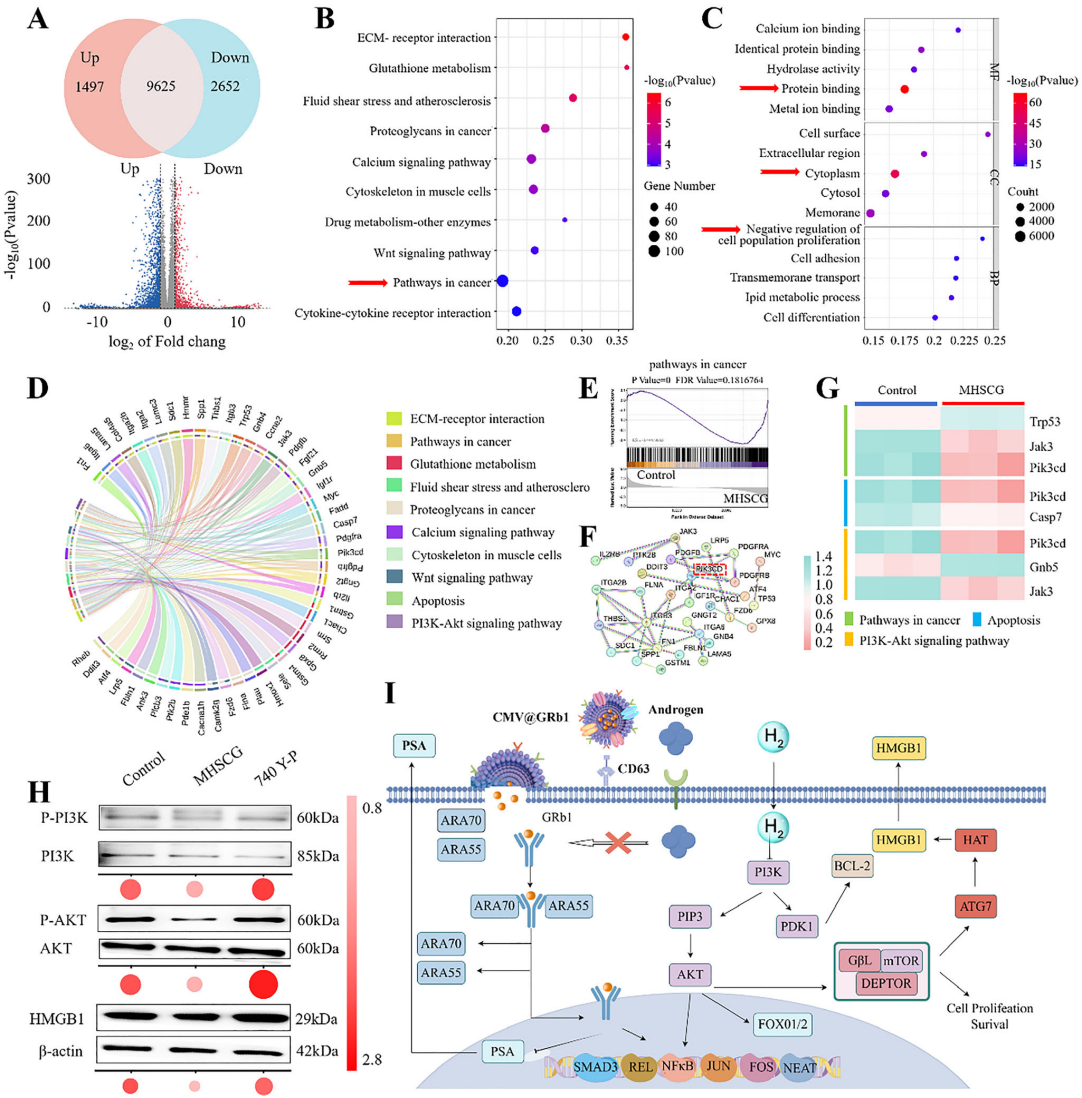
The molecular biological mechanism of MHSCG in prostate cancer cells. A) Volcano plot of gene expression profiles (red: upregulated genes, blue: downregulated genes). B) KEGG enrichment analysis of the top 10 significantly enriched pathways. C) Bubble plot of Gene Ontology (GO) enrichment analysis for the top 15 terms. D) STRING network diagram illustrating gene‐pathway correlations of the top 50 differentially expressed genes. E) Gene Set Enrichment Analysis (GSEA) of pathways in cancer. F) Protein–protein interaction (PPI) network analysis based on the 50 genes of interest. G) Heatmap of gene expression profiles in different treatment groups. H) Western blot analysis of protein expression levels of p‐PI3K, PI3K, p‐AKT, AKT, and HMGB1 in RM‐1 cells. I) Schematic representation of the signaling pathways mediated by MHSCG in prostate cancer cells.

To elucidate the potential therapeutic mechanism of MHSCG on PCa, we conducted a whole‐transcriptome RNA sequencing analysis of RM‐1 cells treated with MHSCG and the untreated group. Differentially expressed genes (DEGs) were identified based on statistical significance (P< 0.05) and fold change criteria (FC >2 or FC< 0.5). Subsequent differential expression analysis between the MHSCG‐treated and Control groups revealed 4149 DEGs, with 1497 genes significantly up‐regulated (P< 0.05 and FC >2) and 2652 genes significantly down‐regulated (P< 0.05 and FC< 0.5), as depicted in the volcano plot in **Figure** **8**A. The Kyoto Encyclopedia of Genes and Genomes (KEGG) pathway analysis of the DEGs indicated a notable enrichment of biological processes related to “pathways in cancer” (Figure 8B). Additionally, the gene ontology (GO) analysis revealed that these DEGs are involved in various biological processes such as protein binding, cytoplasm, and negative regulation of cell population proliferation (Figure 8C). The results of this analysis were presented in Figure 8D, where a circular plot illustrates ten major enriched pathways involving 50 genes of interest (GOIs), such as “Pathways in cancer”, “apoptosis”, and “PI3K‐Akt signaling pathways”. Gene set enrichment analysis (GSEA) confirmed the involvement of “pathway in cancer” in the therapeutic mechanism of MHSCG, indicating its inhibition of PCa cells (Figure 8E). Furthermore, the functional relationships among proteins encoded by these GOIs were elucidated through protein‐protein interaction (PPI) networks, as shown in Figure 8F. Key proteins such as Trp53, Pik3cd, Casp7, and Jak3 were identified to play crucial roles in the mechanism of action of MHSCG and exhibited extensive interactions with other GOIs. Following MHSCG intervention, heatmap visualizations of significant DEGs revealed the downregulation of genes associated with “Pathways in cancer” (e.g., Jak3, Pik3cd), “apoptosis” (e.g., Pik3cd, Casp7), and the “PI3K‐Akt signaling pathway” (e.g., Pik3cd, Jak3) with upregulation of tumor suppressor gene Trp53 associated with tumor immune escape (Figure 8G).^[^ ^41^ ^]^

Based on the predicted gene results, protein immunoblotting was performed to verify the treatment signaling pathways. Results indicated that the MHSCG reduced PI3K and AKT gene phosphorylation while promoting HMGB1 release compared to the Control group.^[^ ^42^ ^]^ Subsequently, co‐treatment of RM‐1 cells with PI3K activator and the MHSCG resulted in elevated HMGB1 expression, confirming that the MHSCG promotes HMGB1 release through inhibition of the PI3K‐AKT signaling pathway (Figure 8H), thereby exerting anti‐tumor effects. A potential therapeutic molecular mechanism diagram was constructed based on these verification results (Figure 8I).

## 3 Conclusion

In conclusion, we synthesized a multifunctional Mg‐based therapeutic platform (MHSCG) that combines the stability of the Mg substrate with spatiotemporal response multi‐mode therapy. This platform featured a bilayer structure of cell membrane condensation, which enhanced the effective linkage of disulfide bonds in HA‐SH through optimized weak hydrophobic interactions. Additionally, it mitigated the corrosion effects of corrosive ions on the Mg substrate. Regarding drug delivery, the HA‐SH and CMV‐modified drug carrier demonstrated intelligent responsiveness to GSH and pH in the tumor microenvironment. This allowed for the cascade release of drugs and facilitated efficient entry into the tumor cytoplasm via the CMV‐mediated membrane fusion mechanism. These modifications significantly enhanced the targeted therapeutic efficacy of drugs. Mechanistic studies indicated that the MHSCG platform, through the release of ginsenoside Rb1 and hydrogen, enabled multi‐modal synergistic therapy involving androgen deprivation therapy (ADT) and immunogenic cell death (ICD). This was achieved by inhibiting the PI3K‐AKT signaling pathway, leading to both androgen receptor antagonism and immune cell activation. This innovative therapeutic platform introduced a novel research paradigm for degradable implants in tumor therapy, enhancing therapeutic outcomes while minimizing structural failure risks, which held significant clinical value and translational potential.

## 4 Experimental Section

Please see the Supporting Information for the experimental sections. Animal experimental protocols were approved by the Animal Ethics Committee of Fudan University (License number: 2025‐YRIFU‐002).

Statistical analyses were conducted using GraphPad Prism software, with results presented as mean ± SD. Student's *t*‐test was employed for comparing two groups, while one‐way analysis of variance (ANOVA) with Tukey post hoc test was used for multiple comparisons. Statistical significance was determined with significance levels denoted as ^*^ *p*< 0.05, ^**^ *p*<0.01, ^***^ *p*< 0.001.

## Conflict of Interest

The authors declare no conflict of interest.

## Supporting information



Supporting Information

Supplemental Movie 1

## Data Availability

The data that support the findings of this study are available from the corresponding author upon reasonable request.
