# Supplementary material for: Magnesium Structure‐Function Integration Platform for Spatiotemporal Multi‐Modality Therapy: Combining Hormonotherapy and Immunotherapy in Prostate Cancer
Source: Adv Sci (Weinh). 2025 Dec 23;13(12):e15235. doi: 10.1002/advs.202515235 (PMC12948271; doi:10.1002/advs.202515235)
Supplement: Supplementary file 1 — Supporting Information [file ADVS-13-e15235-s001.docx]

Supporting Information

Magnesium Structure-Function Integration Platform for Spatiotemporal Multi-Modality Therapy: Combining Hormonotherapy and Immunotherapy in Prostate Cancer

Rui Zan, Qianping Mao, Keyi Wang, Guodong Zou, Shi Yang, Hua Qiu, Xiang Fang, Guiqing Wang, Xinyi Zhou, Jiexia Wen, Shuai Jiang, Ran Huang*, Qiuming Peng*, Tao Suo*

**1. Materials and methods**

**1.1 Instruments**

The morphology was characterized using scanning electron microscopy (SEM; Quanta 450 FEG, Thermo Fisher Scientific, USA) and transmission electron microscopy (TEM; Thermo Fisher Scientific, USA). Atomic force microscopy (AFM; Dimension FastScan, Bruker, Germany) was employed for 3D profiling. Dynamic light scattering (DLS) and zeta potential measurements were conducted using a Zetasizer Nano-ZS (Malvern Instruments, USA). pH monitoring was carried out with a pH meter (FE22-Meter, Mettler Toledo, Switzerland). X-ray photoelectron spectroscopy (XPS) analysis was performed using an ESCALab220i-XL instrument (Thermo Fisher Scientific, USA). Raman spectroscopy was conducted with an R200-L Raman spectrometer (Bruker, Germany). Electrochemical testing was performed using an electrochemical workstation (PGSTAT302N, Autolab, Switzerland). Scanning vibrating electrode technique (SVET) measurements were taken with a VersaSCAN scanning electrochemical workstation (PGSTAT302N, AMETEK, USA). Cell viability was assessed using a microplate reader (Infinite E Plex, Tescan, Switzerland). Flow cytometry analysis was carried out on a CytoFLEX flow cytometer (Beckman, USA). Confocal laser scanning microscopy (CLSM) images were acquired with a Confocal Laser Scanning Microscope-TCS SP8 STED 3X (Leica, Germany). *In vivo* imaging of live mice was conducted using an IVIS Lumina imaging system (PerkinElmer, USA). The concentration of GRb1 and RNA was quantified using NanoDrop and Agilent 2100 bioanalyzer (Thermo Fisher Scientific, USA). The Western blot images were obtained by Amersham Imager 600 (General Electric Co., Ltd., USA).

**1.2 Cell lines and animals**

RM-1 cells were purchased from the Hangzhou Baidi Biotechnology Co, Ltd (China). The mice we used were BALB/C nude mice and C57BL/6J mice (over 18 g) that were acquired from the Center for Experimental Animals, Fudan University (Shanghai, China).

**1.3 Network pharmacological analysis**

The ginsenosides were obtained through TCM System Pharmacology (TCMSP) (http://ibts.hkbu.edu.hk/LSP/tcmsp.php) and their compound names and molecular structures were validated via the PubChem database (https://pubchem.ncbi.nlm.nih.gov/). Screening criteria included an oral bioavailability (OB) of ≤ 8% and a drug-likeness (DL) of ≥ 0.18. Subsequently, potential targets of the ginsenosides were identified and consolidated using the TCMSP analysis platform, with all target names confirmed through the UniProt database (https://www.uniprot.org/). Utilizing the GeneCards database (https://www.genecards.org/), a search was conducted using the keyword “prostate cancer” to compile and integrate all relevant genes. The overlap between the targets of ginsenosides, prostate cancer (PCa) and immunology yielded the therapeutic targets through a Venn diagram search. A network diagram illustrating the active components of ginsenosides was generated using Cytoscape 3.2.1 software, followed by an analysis of the network’s topological parameters and the assessment of node significance based on centrality and node degree using Cytoscape’s network analysis tool. Therapeutic targets and protein-protein interaction (PPI) networks were established utilizing the STRING database (https://string-db.org/), with the parameters set at a “minimum required interaction score = 0.7” and “hide disconnected nodes in the network”. Gene Ontology (GO) and Kyoto Encyclopedia of Genes and Genomes (KEGG) pathway enrichment analyses were conducted using the David v6.8 database (https://david.ncifcrf.gov/). Lastly, molecular docking was employed to examine the interaction between ginsenosides and androgen receptors.

**1.4 Preparation of CMV**

CMV were isolated using differential ultracentrifugation. Following the culture of 20-50 million cells, cells were collected and centrifuged at 600 g for 5 min at 4 ℃ to obtain cell pellets. The pellets were then treated with 1 mL of membrane protein extraction reagent A (Beyotime, China) containing PMSF (Beyotime, China). After ice bath treatment for 15 min, the mixture was transferred to a pre-cooled glass homogenizer and homogenized approximately 50 times. Subsequently, the homogenate was centrifuged at 700 g for 10 minutes at 4 ℃. The supernatant was carefully collected into new centrifuge tubes and further centrifuged at 14,000 g for 30 min at 4 ℃ to precipitate cell membrane fragments.

**1.5 Preparation of CMV-GRb1**

The mass of CMV was quantified using a BCA protein detection kit (Servicebio, China). The CMV were resuspended in PBS to a concentration of 1 mg/mL and mixed with 0.5 mg/mL GRb1. The mixture was then sonicated in an ice bath for 30 min, and CMV-GRb1 were obtained through a liposome extruder.

**1.6 Preparation of different samples**

The Mg samples were polished with silicon carbide (SiC) abrasive paper of 800, 1500 and 3000 grit, respectively. Then, the Mg samples were cleaned in acetone and ethanol for 15 min to remove residual particulates and contaminants. To obtain an Mg(OH)_2_ coating on the surface of the Mg substrate, we subsequently placed the treated Mg samples with 13 mL of 1 mol/L NaOH solution in reaction vessel at 60 ℃ for 24 h. The resulting product underwent ultrasonic cleaning with ddH_2_O for 5 min, a process that was repeated three times. Then, the Mg(OH)₂ samples were immersed in HA-SH solution, a mixture of HA-SH and GRb1 (mass ratio 1:10), and a mixture of HA-SH and CMV-GRb1 (mass ratio 1:10). The samples were then incubated at 37 °C for 6 h to yield MHS, MHSG, and MHSCG samples, respectively.

**1.7 Responsiveness of MHSCG**

The responsiveness of MHSCG was assessed by immersing samples in artificial solutions with varying concentrations of GSH (0 mM, 2 mM, 10 mM) and incubating them at 37 ℃ with agitation. The concentration of GRb1 in the immersion solution was quantified using UV spectrophotometry at 4 h, 12 h, and 24 h time intervals. Additionally, the pH responsiveness of CMV@GRb1 was evaluated by immersing it in solutions with different pH levels (5.4, 6.4, 7.4) and incubating at 37 ℃ with shaking. The concentration of GRb1 was measured at 4 h, 12 h, and 24 h time points.

**1.8 Corrosion testing**

The MHSCG specimens underwent corrosion testing by immersion in artificial urine at 37 ℃, with a consistent ratio of 20 mL/cm^2^ of artificial urine volume to sample surface area. The pH value of the solution was monitored daily throughout the immersion period. Weight loss assessments were performed over a 14 d immersion test. To monitor the H_2_ release, the MHSCG samples were immersed in 11 mL of artificial urine solution, with the evolved H_2_ collected via an inverted funnel and quantified using a calibrated burette.

The electrochemical properties of the different samples were measured using an electrochemical workstation, with an exposed area of 1.2 cm^2^ served as the working electrode in artificial urine solution. In addition, the corrosion behavior of the specimens was evaluated by the SVET measurements. The specimens immersed in solution were laterally scanned after 0 h, 12 h and 24 h. The vertical volta potential was recorded at a height of about 100 µm from surface by a probe tip (Pt–Ir, 10 µm) on a lattice of 21 × 21 points over an area of 3 × 3 mm^2^ (each step size of 150 µm, a measuring speed of 150 µm/s).

**1.9 Fluid dynamics simulations**

We employed first-principles calculations based on density functional theory (DFT) using the Vienna Ab initio Simulation Package (VASP) to investigate the adsorption energy of sodium hyaluronate on the (001) plane of Mg(OH)_2_. The exchange and correlation interactions between electrons were described using the Perdew-Burke-Ernzerhof (PBE) functional within the Generalized Gradient Approximation (GGA). A plane-wave cutoff energy of 500 eV was set to ensure accurate and convergent results. The convergence criteria for total energy and force components were set to 1×10^-^⁵ eV and 0.05 eV/A, respectively. A Monkhorst-Pack k-point grid with a density of 1×1×1 was used to sample the reciprocal space for geometry optimization.

**1.10 Molecular dynamics simulation**

We employed the molecular dynamics method using the LAMMPS software based on the Lennard-Jones (L-J) approach, to simulate the diffusion process of Cl^-^ and sodium hyaluronate (HS) on the surface combined with Mg(OH)_2_ and HS at conditions of 25 °C. The equation for L-J potential is as follows:

$$\text{E}\text{=4}\text{ϵ}\text{[}{\text{(}\frac{\text{σ}}{\text{r}}\text{)}}^{\text{12}}\text{- }{\text{(}\frac{\text{σ}}{\text{r}}\text{)}}^{\text{6}}]$$

Here, *ϵ* represents the coefficient of force intensity, *σ* is the bond length, and *r* is the distance between two atoms.

**1.11 Cellular uptake and lysosomal escape detection**

RM-1 cells were incubated with coumarin 6-labeled CMV-GRb1 at 37 ℃ for 24 h, washed twice with PBS, and then incubated with lysosomal red fluorescent probe at 37 ℃ for 2h. After PBS washing, cells were fixed with 4% paraformaldehyde (Servicebio, China) for 10 min, stained with 20 μL DAPI (Beyotime, China) for 5 min at room temperature, washed twice with PBS, and photographed using CLSM.

**1.12 *In Vitro* cytotoxicity testing**

MHSCG was immersed in 1.884 mL complete culture medium for 3 d. Then, the extract was added to 96-well plates seeded with RM-1 cells. Cell viability was detected using Cell Counting Kit-8 (Dojindo, Japan) after 24 h of culture and quantified with a microplate reader.

**1.13 Cell apoptosis**

RM-1 cell apoptosis induced by MHSCG was assessed by seeding the cells in 6-well plates followed by a 24 h incubation with extract. The collected cells were then stained with fluorescein (FITC) annexin V and propidium iodide (PI) solution, as well as PI/RNase staining buffer (Beyotime, China). Flow cytometry analysis was performed, and the data were processed using FlowJo software (BD, USA).

**1.14 3D spheroid experiments**

For 3D tumor spheroid growth, RM-1 cells were seeded in ultra-low attachment round-bottom 96-well plates. Samples were incubated under standard culture conditions for a total of 7 d. Subsequently, cultures were treated with extract. Subsequently, cells were washed with PBS and the cells were further incubated with calcein-AM and propidium iodide for 30 min. Finally, the therapeutic effect of various formulas was visualized by CLSM.

**1.15 Maturation of dendritic cells**

The RM-1 cells were co-cultured with the DCs in transwell plate for 24 h. After this time, DC were stained with the corresponding antibody (PE anti-mouse CD11c antibody, FITC anti-mouse CD80 antibody, APC anti-mouse CD86 antibody, Biolegend, USA) for 1 h. The maturation of the dendritic cells was assessed by flow cytometry.

**1.16 *In vivo* cancer treatment efficacy**

Six-week-old male BALB/C nude mice (18 ± 2g) were used as animal models. For tumor inoculation, 200 μL of RM-1 cell solution (PBS buffer, 5×10^6^ cells) was subcutaneously injected under the armpit of each mouse to establish the tumor model. Tumor size and mouse weight were monitored every two days. Tumor volume was calculated using the formula:

tumor volume =$\frac{L\times W^{2}}{2}$

where *L* is the length of the tumor, and the *W* is the width of the tumor.

Following a 14 d treatment period, the tumors and major organs (heart, liver, spleen, lung, and kidney) were harvested post-sacrifice of the mice. Subsequently, TUNEL and caspase 3 staining were performed on the tumors, while the organs underwent H&E staining to assess in vivo biocompatibility.

**1.17 The immunotherapy *in vivo***

C57BL/6J mice were injected subcutaneously with RM-1 cells to establish a tumor model. On the 9 d post-injection, the mice were euthanized, and tumor tissues along with adjacent lymph nodes were harvested. Three tumor samples and lymph nodes were processed by homogenization in FACS buffer, enzymatic digestion, and filtration through nylon gauze to obtain single-cell suspensions. These cells underwent staining with fluorescence-labeled antibodies for the following analyses: 1) dendritic cell (DC) maturation (lymph nodes single-cell suspensions stained with Fc blocker (anti-mouse CD16/32), anti-mouse CD11c, anti-mouse CD80, and anti-mouse CD86); 2) CD8^+^/CD4^+^ T cell populations (tumor single-cell suspensions stained with Fc blocker (anti-mouse CD16/32), anti-mouse CD3ε, anti-mouse CD8a, and anti-mouse CD4); 3) macrophage polarization (tumor single-cell suspensions stained with Fc blocker (anti-mouse CD16/32), anti-mouse CD11b, anti-mouse F4/80, anti-mouse CD206, and anti-mouse CD86); 4) regulatory T (Treg) cell analysis (tumor single-cell suspensions stained with Fc blocker (anti-mouse CD16/32), anti-mouse CD4, anti-mouse CD25, treated with a True-Nuclear kit, and stained with anti-mouse FOXP3). The stained cells were then analyzed using flow cytometry and FlowJo software. Additionally, other tumor samples were fixed in 4% paraformaldehyde, followed by histological staining with H&E and Masson staining.

**1.18 RNA Sequencing**

To investigate the molecular mechanism of tumor cell death induced by MHSCG, RM-1 cells were cultured in wxtract for 24 h. Cells treated with complete culture medium served as the control group. Total RNA was extracted using Trizol (Beyotime, China). Subsequently, total RNA was quantified using NanoDrop and Agilent 2100 bioanalyzer. mRNA was purified using Oligo(dT)-linked magnetic beads. Double-stranded PCR products were heat-denatured and circularized by splint oligonucleotide sequence to obtain the final library. Single-stranded circular DNA was formatted to the final library. For comparison of differentially expressed genes (DEGs) between MHSCG and control groups, data were analyzed using bioinformatics tools, considering genes with fold change values ≥ 2 and P-values < 0.05.

Real-time RT-qPCR assays. The total RNA was extracted from tumor tissue using an RNA-quick purification kit (Simgen, 5003050). Afterward, the total RNA was reverse transcribed into cDNA using HiScript II 1st strand cDNA synthesis kit (Servicebio, G3329-100). The sequences of primers used for RT-qPCR were shown in Table S3. Quantitative real-time PCR was performed using SYBR qPCR master mix (Simgen, 7106500) and Analytikjena qTower 3G.

**1.19 Statistical Analysis**

All data were expressed as mean ± SD, and statistical analysis was performed using Student’s t-test. Statistical significance is denoted by *p < 0.05, **p < 0.01 and ***p <0.001.

**2. Supplementary figures**


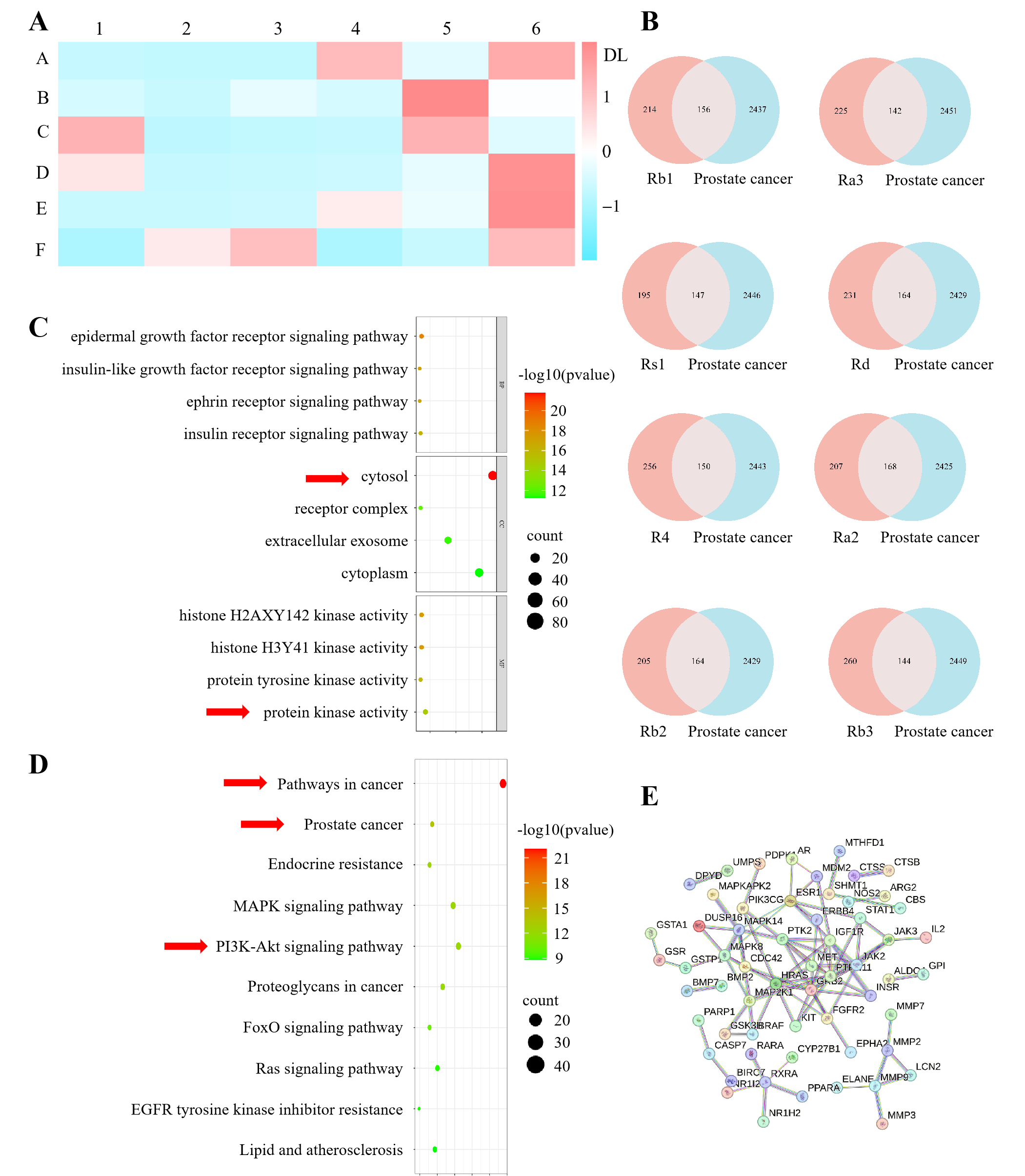


**Figure S1.** Virtual drug screening and potential pathway prediction. (A) Drug similarity screening. (B) Eight ginsenosides and VENN diagram of prostate cancer. (C, D) GO (C) and KEGG (D) enrichment analysis of GRb1 in the treatment of prostate cancer. (E) The PPI network predicts the potential target of GRb1 in the treatment of prostate cancer.


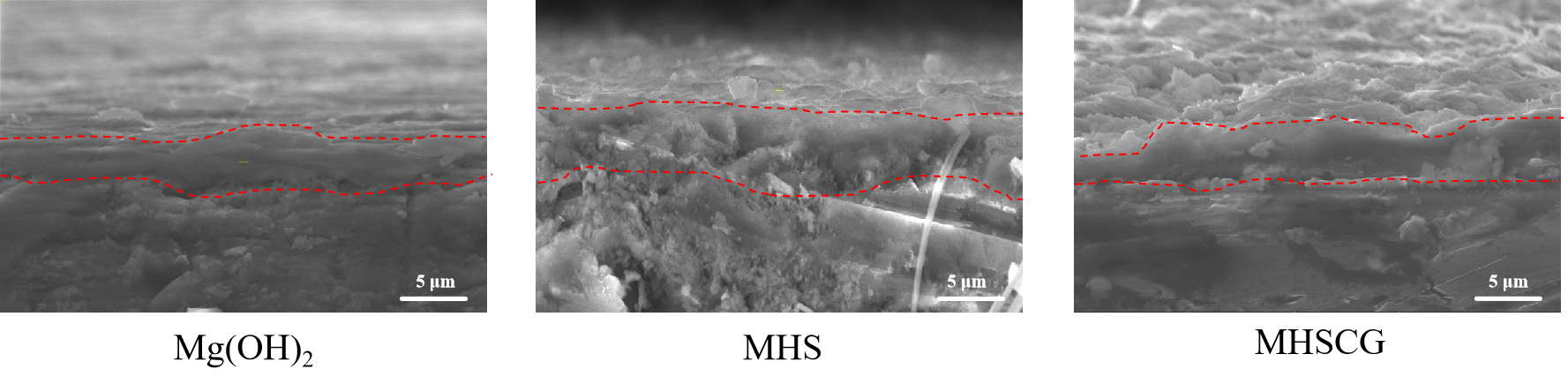
**
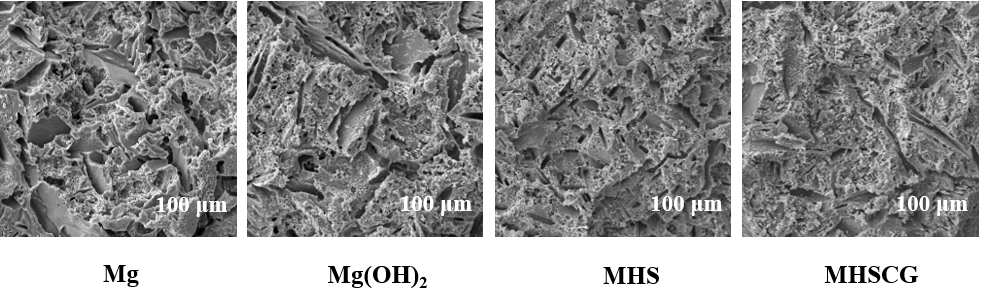
**

**Figure S2.** Coating thickness and degradation surface morphology of Mg(OH)_2_, MHS and MHSCG groups.


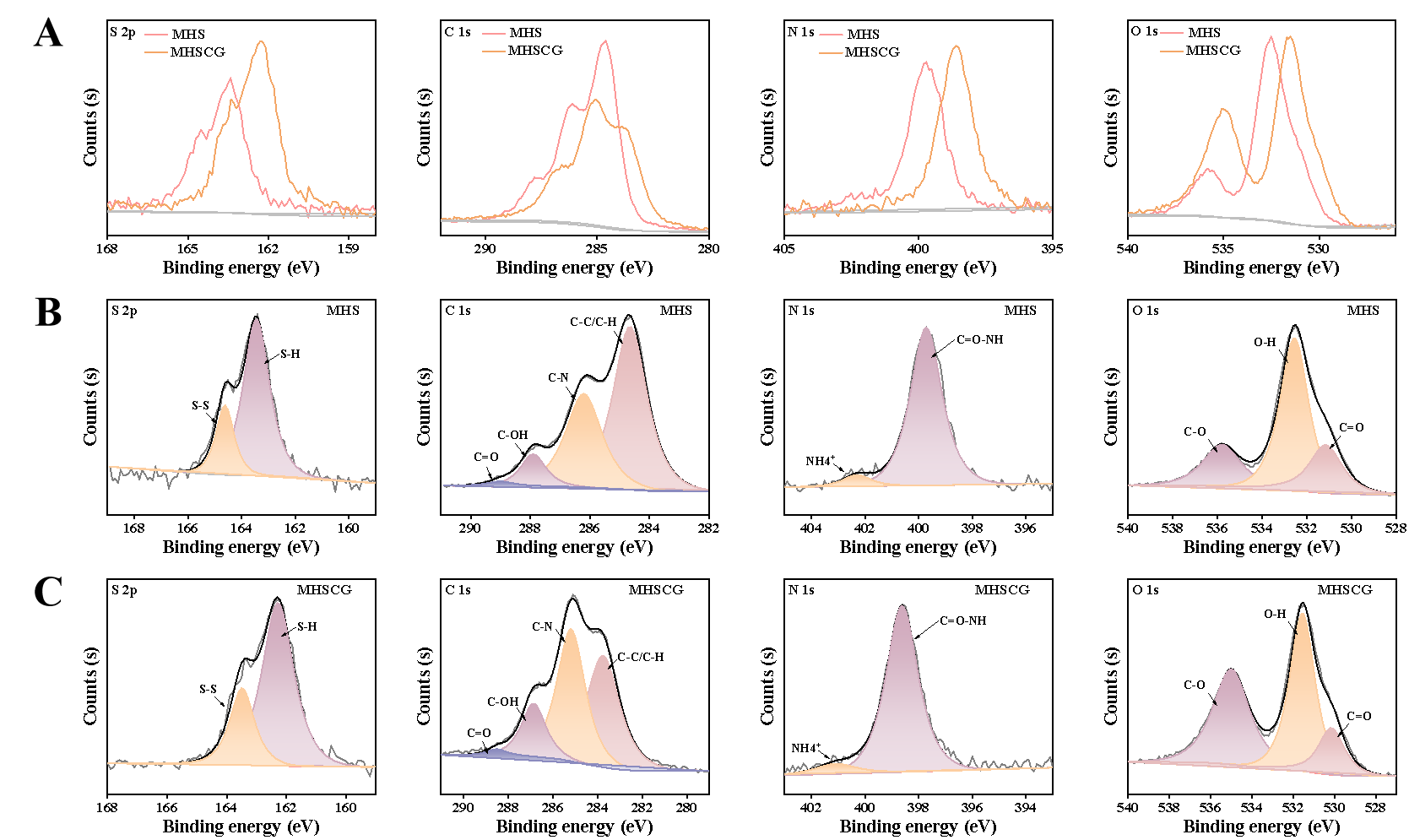


**Figure S3.** XPS results of MHS and MHSCG. (A) The comparison of S 2p, C 1s, N 1s and O 1s between MHS and MHSCG. (B, C) High-resolution S 2p, C 1s, N 1s, and O 1s of MHS (B) and MHSCG (C).


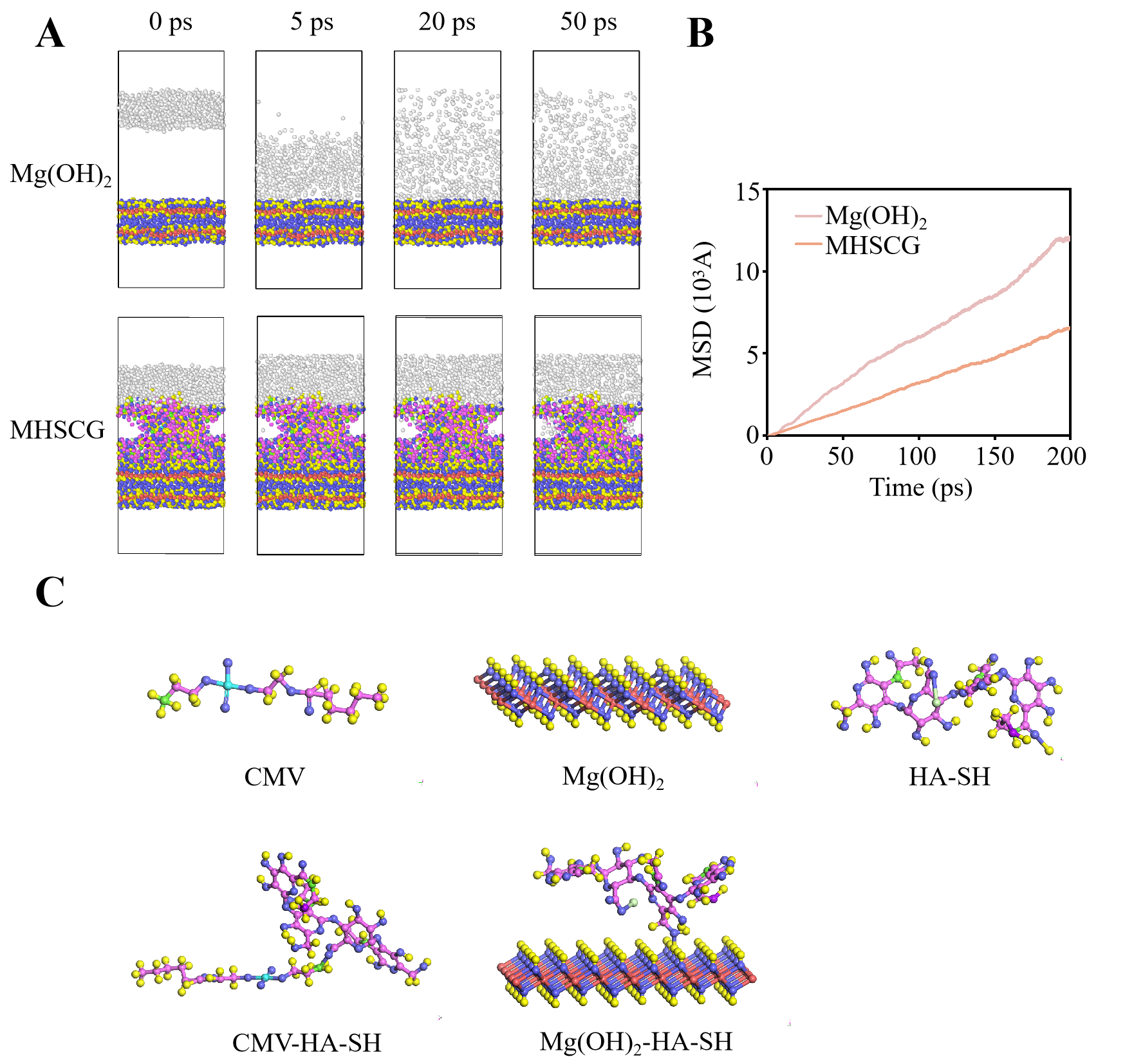


**Figure S4.** The simulation of dynamic chloride ion (Cl^-^) on the surface of Mg(OH)_2_ and MHSCG. (A) Cl^-^ diffusion within the 50 ps. (B) The diffusion curves on the surfaces of Mg(OH)_2_ and MHSCG. (C) The structure of each group.


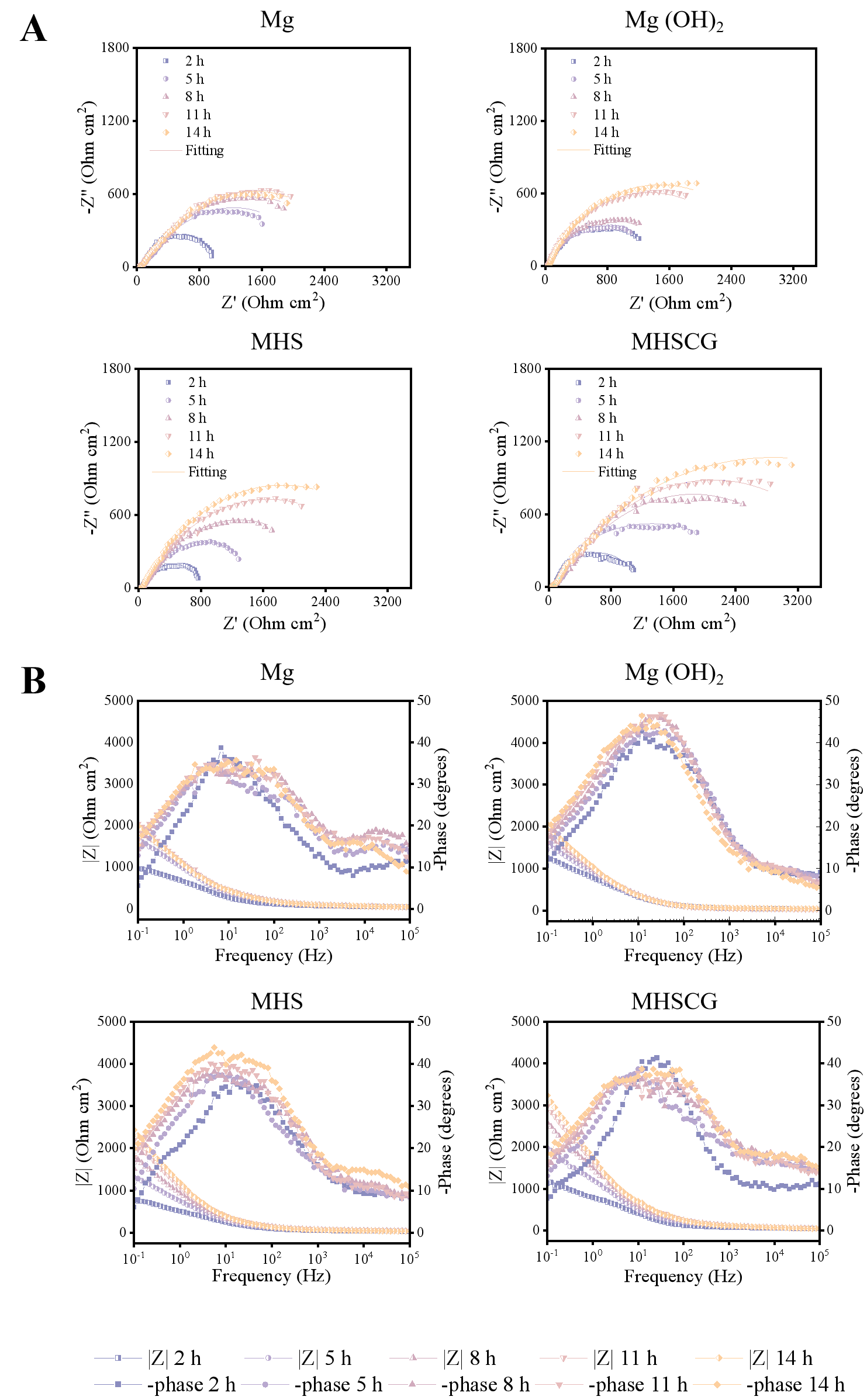


**Figure S5.** Corrosion-resistant electrochemical detection. (A, B) Nyquist curves (A) and Bode curves (B) of Mg, Mg(OH)_2_, MHS and MHSCG at different time points.


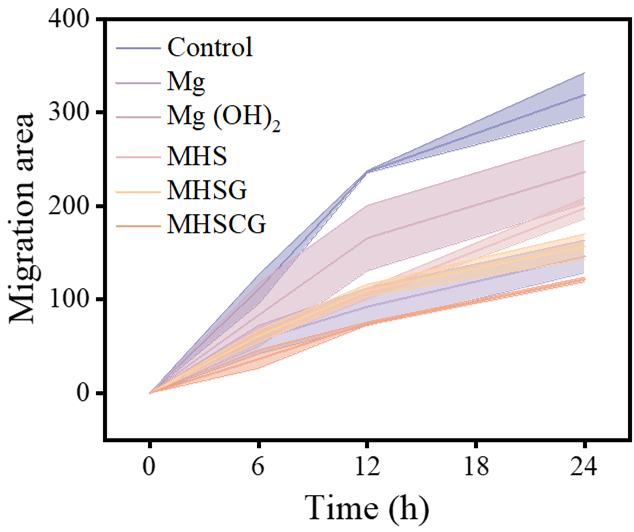


**Figure S6.** Quantitative analysis of cell migration.


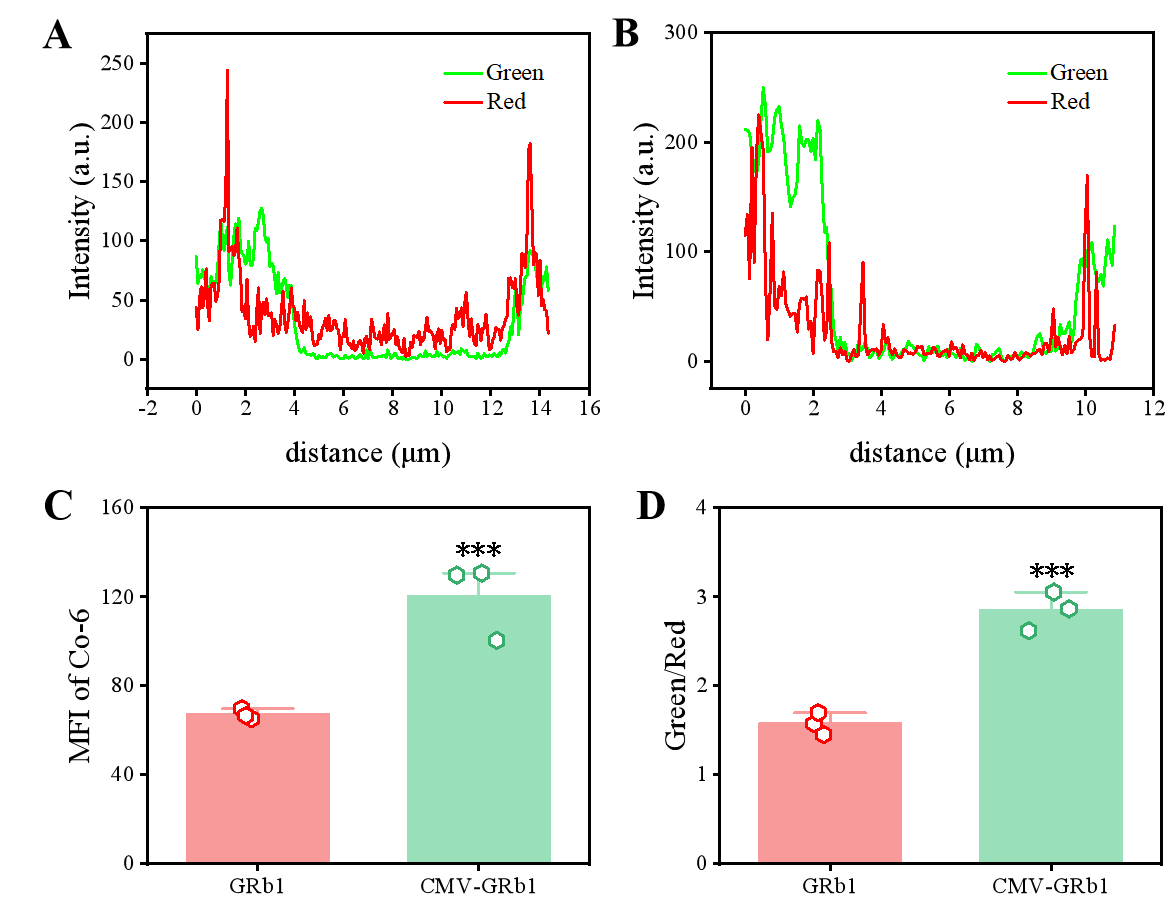


**Figure S7.** Quantitative analysis of cell uptake. (A, B) Fluorescence co-localization analysis of GRb1 (A) and CMV-GRb1 (B). (C) Quantitative intake of GRb1 and CMV-GRb1. (D) Lysosomal fluorescence analysis. ****p* < 0.001 (n ≥ 3).


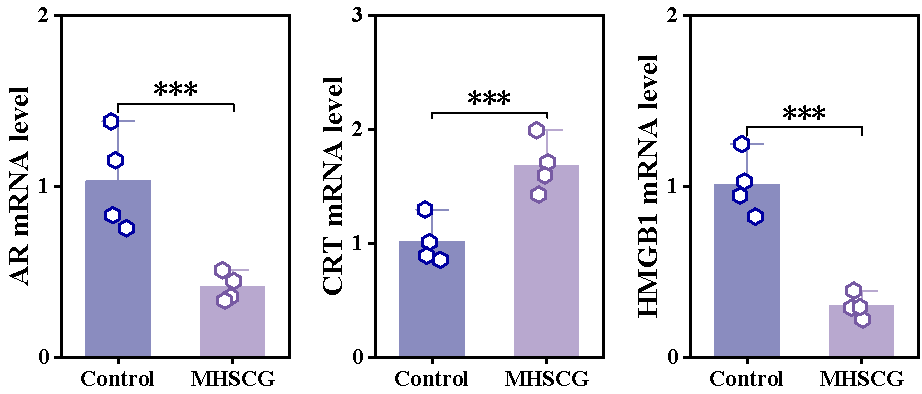


**Figure S8.** RT-qPCR analysis of the expression levels of AR, CRT, and HMGB1 in cancer after treatments. ****p* < 0.001 (n ≥ 3).


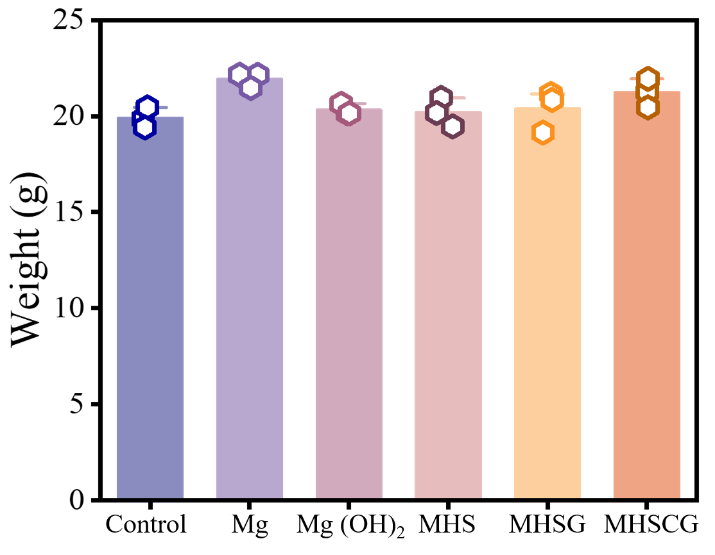


**Figure S9.** The body weight of nude mice after 15 days.


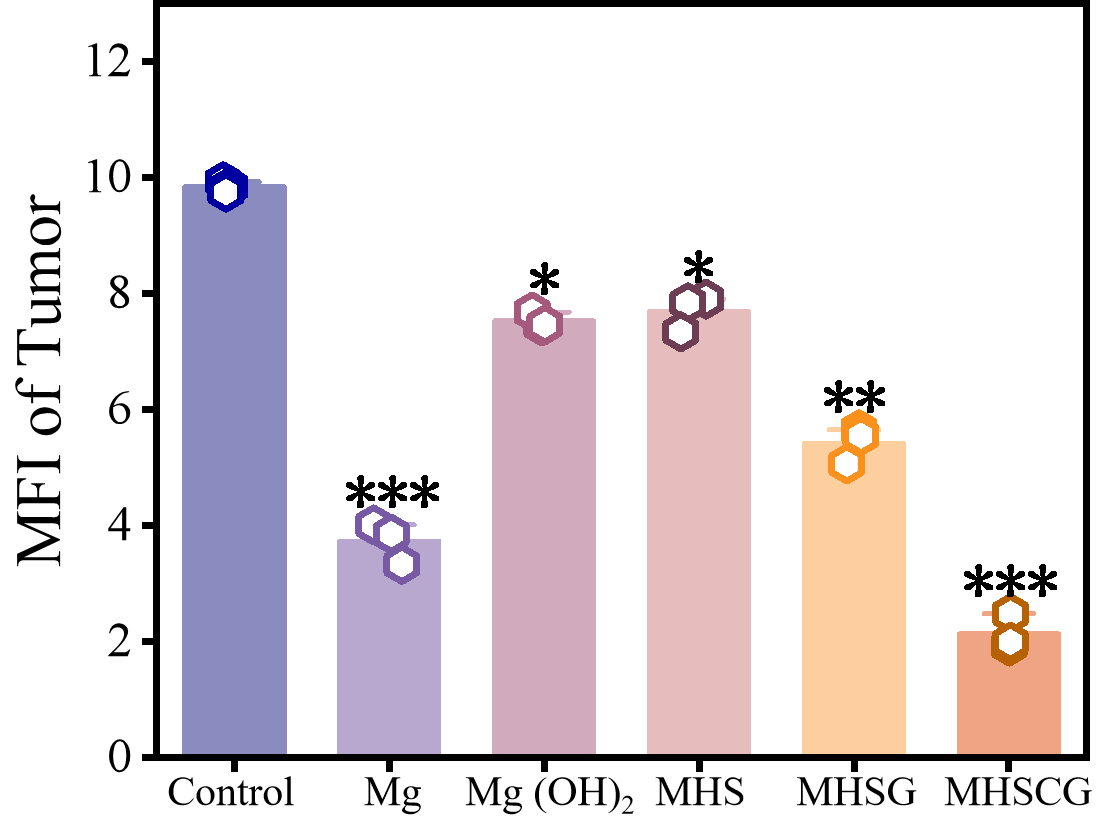


**Figure S10.** Quantitative analysis of in vivo fluorescence intensity of nude mouse tumors. **p* < 0.05, ***p* < 0.01, and ****p* < 0.001 (n ≥ 3).


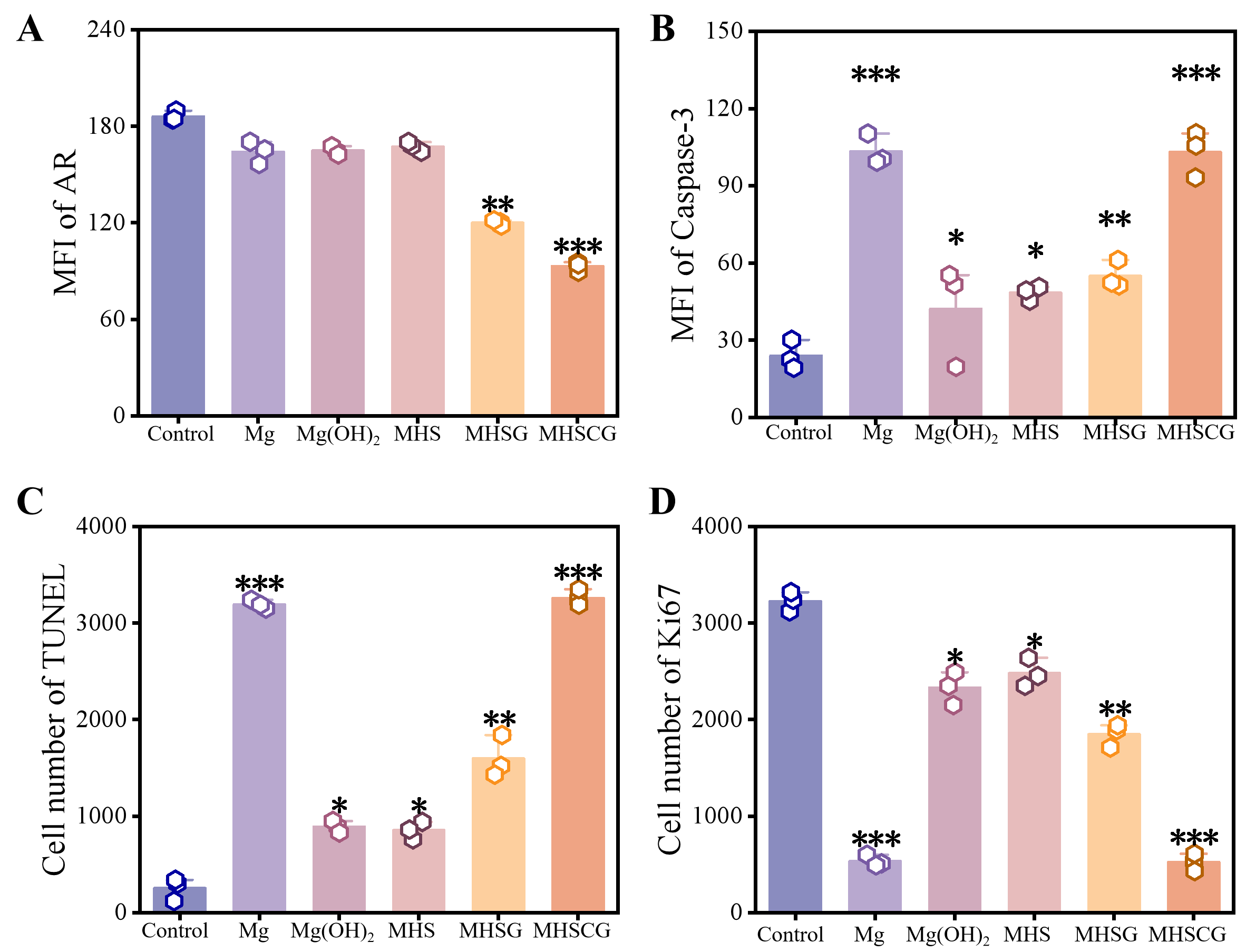


**Figure S11.** The quantification of AR expression and apoptosis in vivo. (A-D) Quantitative analysis of AR (A), Caspase-3 (B), Tunel (C), and Ki67 (D) in tumor tissues of nude mice. **p* < 0.05, ***p* < 0.01, and ****p* < 0.001 (n ≥ 3).


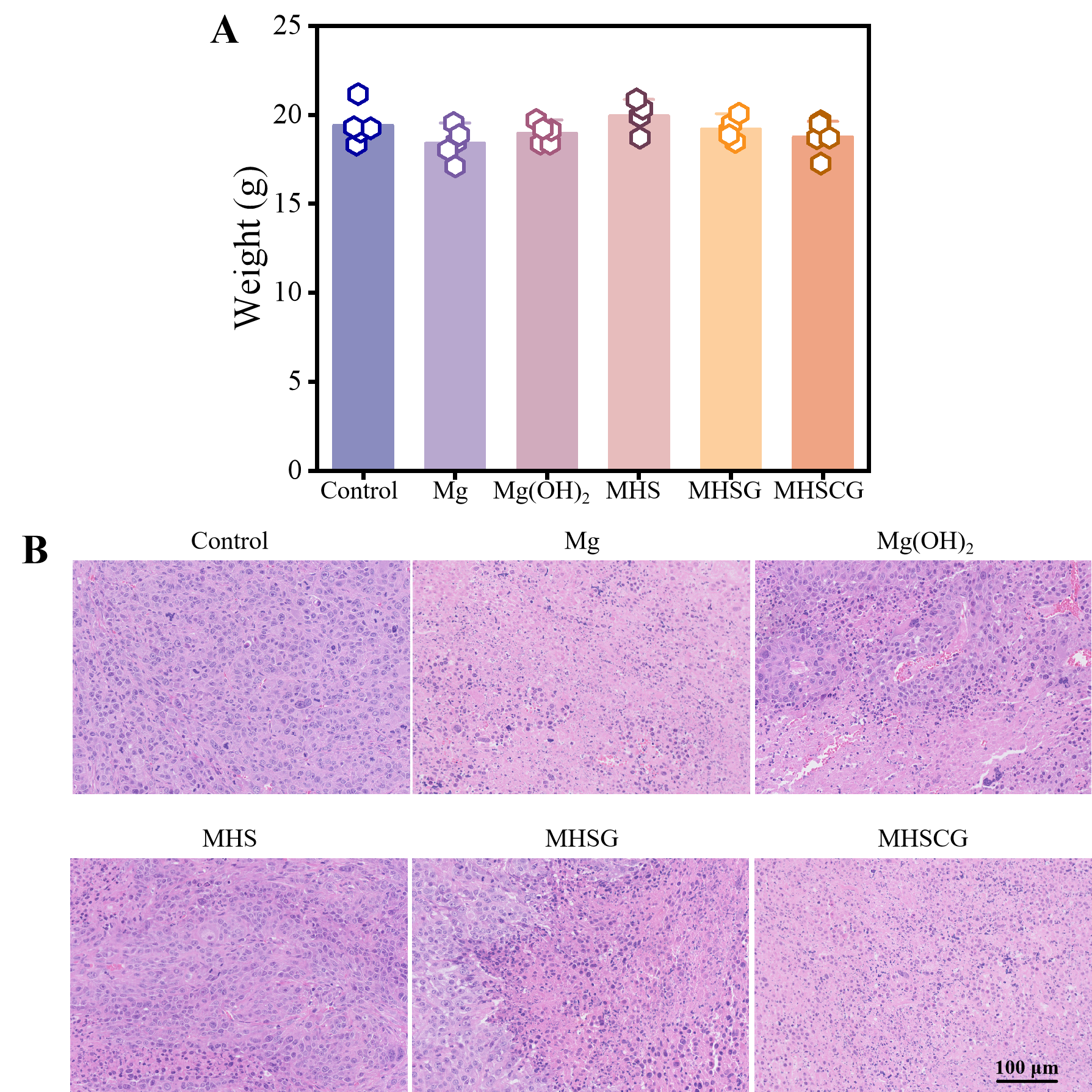


**Figure S12.** Detection of tumor growth inhibition effect.(A) The body weight of C57 mice after 9 d treatment. (B) H&E staining of tumor tissues.


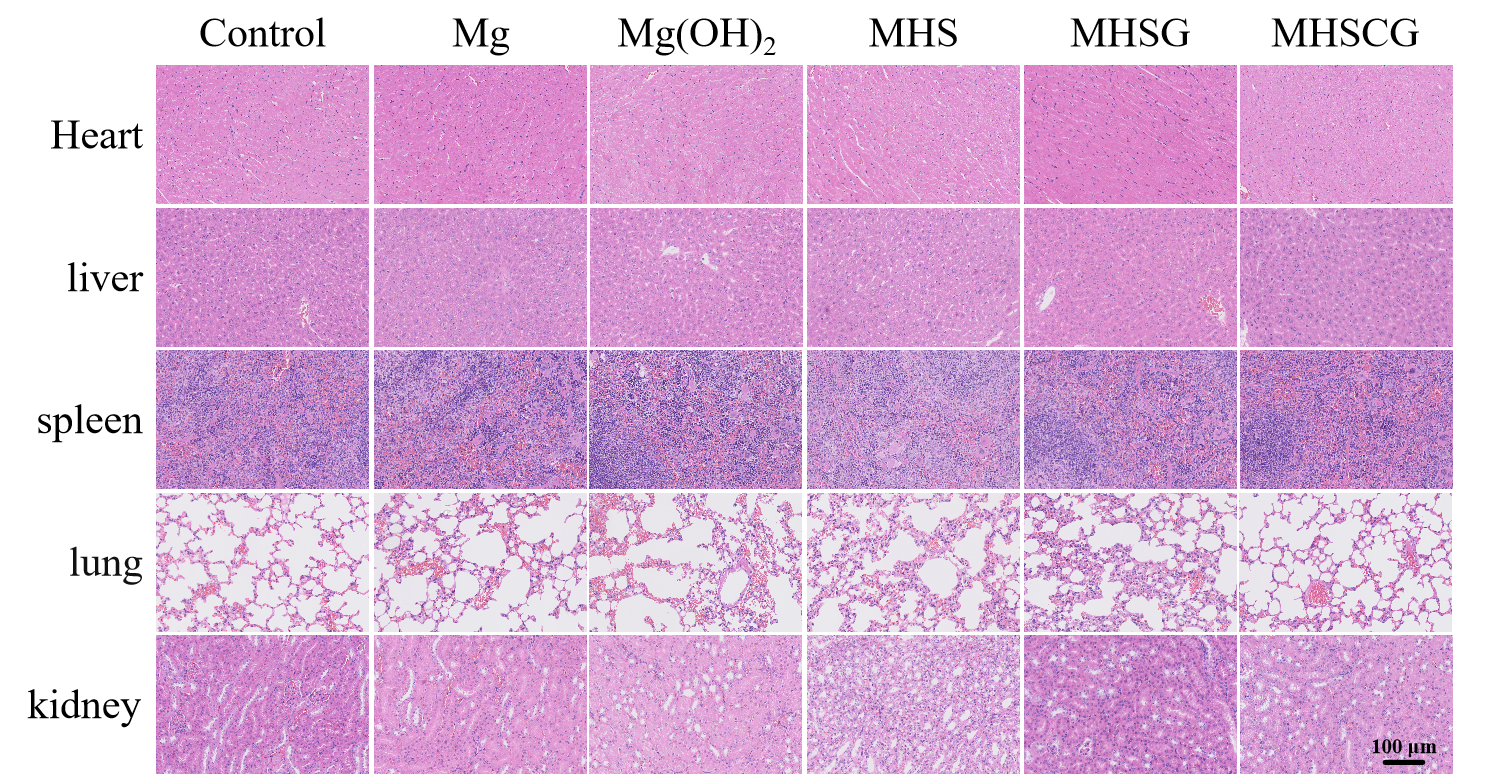


**Figure S13.** H&E staining of heart, liver, spleen, lung and kidney tissues.


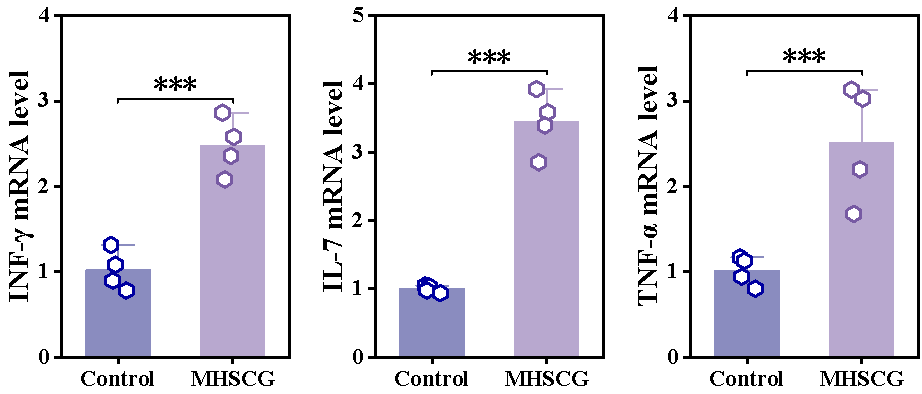


**Figure S14.** RT-qPCR analysis of the expression levels of INF-γ, IL-7, and TNF-α in tumor tissues after treatments. ****p* < 0.001 (n = 4).


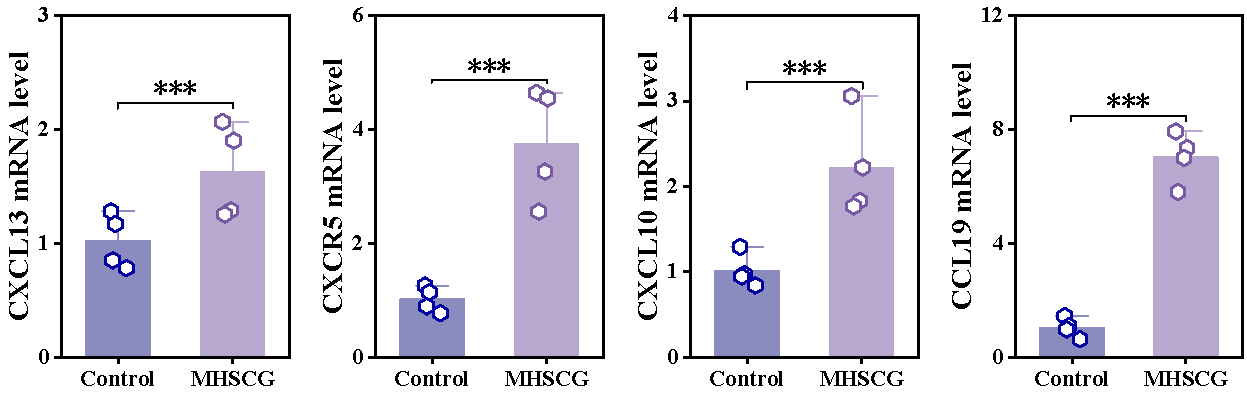


**Figure S15.** RT-qPCR analysis of the expression levels of CXCL13, CXCR5, CXCL10, and CCL19 in tumor tissues after treatments. ****p* < 0.001 (n = 4).


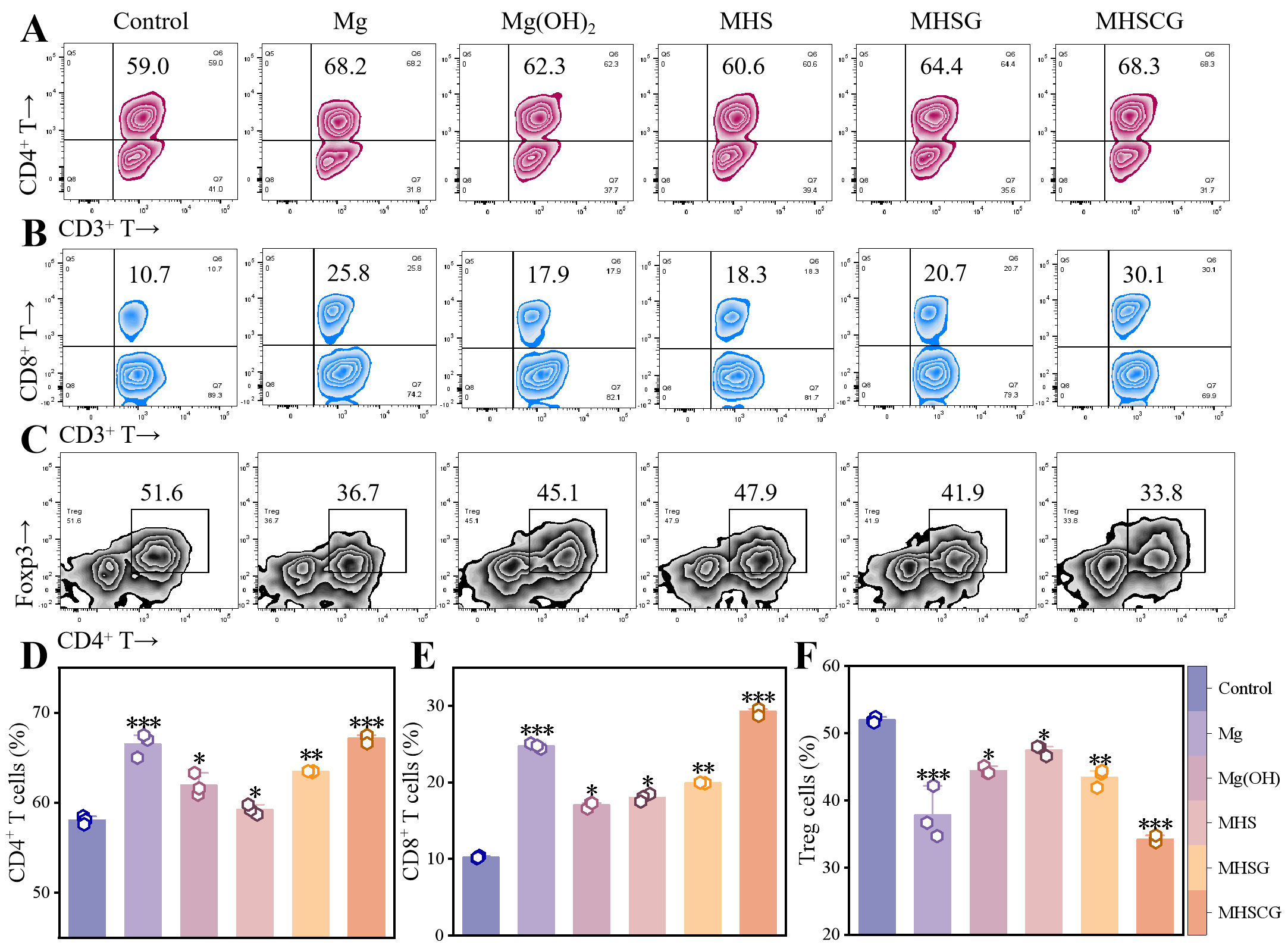


**Figure S16.** (A-C) Flow cytometric analysis of CD4^+^ T cells (A), CD8^+^ T cells (B) and Treg cells (C) in tumor tissues by various treatments. (D-F) Quantitative assessment of CD4^+^ T cells (D), CD8^+^ T cells (E) and Treg cells (F) in tumor tissues based on flow cytometric analysis. **p* < 0.05, ***p* < 0.01, and ****p* < 0.001 (n ≥ 3).

**Table S1.** The fitting results of polarization curves for different samples immersed in artificial. urine for 14 h.

| Samples | *E_corr_*(V_SCE_) | *I_corr_*(A/cm^2^) | *β_a_*(V) | *β_c_*(V) | *P_i_*(mm/y) |
| --- | --- | --- | --- | --- | --- |
| Mg | -1.447 | 1.39×10^-4^ | 0.007 | 0.300 | 3.17 |
| Mg(OH)_2_ | -1.547 | 7.60×10^-5^ | 0.025 | 0.260 | 1.74 |
| MHS | -1.613 | 4.20×10^-5^ | 0.029 | 0.239 | 0.95 |
| MHSCG | -1.525 | 2.41×10^-5^ | 0.047 | 0.212 | 0.55 |

**Table S2.** Fitted EIS results of Mg, Mg(OH)_2_, MHS and MHSCG in artificial urine for different immersion time.

| **Samples** | **Time（h）** | **R_s_(Ohm cm^2^)** | **R_c_(Ohm cm^2^)** | **Q_1_(F·S^n-1^ cm^-2^)** | **R_ct_(Ohm cm^2^)** | **Q_2_(F·S^n-1^ cm^-2^)** |
| --- | --- | --- | --- | --- | --- | --- |
| **Mg** | 2 | 30.3 | 44.1 | 3.15×10^-6^ | 996.0 | 1.73×10^-5^ |
|  | 5 | 28.4 | 58.0 | 2.39×10^-6^ | 2318.5 | 2.08×10^-5^ |
|  | 8 | 32.0 | 32.9 | 5.41×10^-8^ | 2790.4 | 1.97×10^-5^ |
|  | 11 | 27.0 | 62.9 | 4.07×10^-6^ | 2851.9 | 1.46×10^-5^ |
|  | 14 | 34.8 | 44.8 | 3.24×10^-6^ | 3049.0 | 1.80×10^-5^ |
| **Mg(OH)_2_** | 2 | 32.7 | 103.5 | 2.13×10^-5^ | 1352.9 | 3.81×10^-7^ |
|  | 5 | 29.4 | 187.6 | 2.08×10^-5^ | 2096.0 | 5.53×10^-7^ |
|  | 8 | 26.1 | 36.5 | 1.83×10^-5^ | 2473.3 | 2.18×10^-6^ |
|  | 11 | 27.3 | 42.7 | 1.81×10^-6^ | 2731.3 | 1.89×10^-6^ |
|  | 14 | 36.1 | 47.0 | 1.79×10^-5^ | 3001.2 | 2.37×10^-6^ |
| **MHS** | 2 | 23.3 | 33.8 | 2.02×10^-5^ | 862.2 | 1.07×10^-5^ |
|  | 5 | 34.8 | 37.8 | 6.08×10^-6^ | 1545.6 | 1.69×10^-5^ |
|  | 8 | 32.1 | 23.0 | 9.21×10^-7^ | 2479.7 | 2.13×10^-5^ |
|  | 11 | 32.4 | 46.5 | 1.08×10^-5^ | 3313.7 | 8.77×10^-6^ |
|  | 14 | 22.0 | 52.6 | 1.10×10^-5^ | 3722.5 | 7.12×10^-6^ |
| **MHSCG** | 2 | 38.4 | 99.2 | 1.87×10^-5^ | 1171.0 | 8.62×10^-5^ |
|  | 5 | 34.8 | 187.6 | 1.18×10^-5^ | 2275.5 | 3.74×10^-6^ |
|  | 8 | 32.0 | 77.0 | 2.18×10^-6^ | 3521.4 | 1.17×10^-5^ |
|  | 11 | 31.5 | 67.3 | 2.62×10^-6^ | 4048.8 | 9.35×10^-6^ |
|  | 14 | 23.7 | 144.5 | 1.23×10^-5^ | 5949.3 | 2.20×10^-6^ |

**Table S3.** The sequences of primers used for RT-qPCR

| **Target mRNA** | **Forward primer** | **Reverse primer** |
| --- | --- | --- |
| AR | TAGGGCTGGGAAGGGTCTAC | CTATGTTAGCGGCCTCAGGG |
| HMGB1 | GTTCTGAGTACCGCCCCAAA | CTGCTGCAGTGTTGTTCCAC |
| CRT | CCTGAATACTCCCCCGATGC | ACTCCTCTGCATAGGCCTCA |
| INF-γ | GGAACTGGCAAAAGGATGGTGAC | TGACGCTTATGTTGTTGCTGATGG |
| IL-7 | CTGCTCGCAAGTTGAAGCAA | TCACCAGTGTTTGTGTGCCT |
| TNF-α | ATGGCCTCCCTCTCATCAGT | AAGGTACAACCCATCGGCTG |
| CXCL13 | CTCTCCAGGCCACGGTATTC | CCAGGGGGCGTAACTTGAAT |
| CCL19 | TTCAGCCTGCTGGTTCTCTG | ACAGACAGGCAGCAGTCTTC |
| CXCL10 | TTTCTGCCTCATCCTGCTGG | CATTCTCACTGGCCCGTCAT |
| CXCR5 | TCCTAGGGACCTTCCTCTGC | GTGCAGGTGATGTGGATGGA |
| β-actin | TACTGCTCTGGCTCCTAGCA | CGGACTCATCGTACTCCTGC |
